# Wild *Vanilla* and pollinators at risk of spatial mismatch in a changing climate

**DOI:** 10.3389/fpls.2025.1585540

**Published:** 2025-07-03

**Authors:** Charlotte Watteyn, Tobias Fremout, Adam P. Karremans, Koenraad Van Meerbeek, Steven B. Janssens, Sander de Backer, Monika M. Lipińska, Bart Muys

**Affiliations:** ^1^ Department of Earth and Environmental Sciences, KU Leuven, Leuven, Belgium; ^2^ Centro de Investigación Jardín Botánico Lankester, Universidad de Costa Rica, Cartago, Costa Rica; ^3^ Meise Botanic Garden, Meise, Belgium; ^4^ KU Leuven Plant Institute, KU Leuven, Leuven, Belgium; ^5^ Bioversity International, Lima, Peru; ^6^ Department of Biology, KU Leuven, Leuven, Belgium; ^7^ Department of Plant Taxonomy and Nature Conservation, Faculty of Biology, University of Gdańsk, Gdańsk, Poland; ^8^ Instituto de Investigación en Ciencias Naturales y Tecnología (Iarna), Universidad Rafael Landívar, Ciudad de Guatemala, Guatemala

**Keywords:** climate change, Euglossini, *ex situ* conservation, *in situ* conservation, Orchidaceae, plant-pollinator decoupling, species distribution models, vanilla crop wild relatives

## Abstract

Climate change is expected to drive substantial shifts in species’ geographic ranges. Species-specific responses of interacting species, such as plants and their pollinators, may lead to a spatial mismatch in their future distributions, disrupting these interspecific interactions. The crop wild relatives (CWRs) of the tropical cash crop vanilla hold valuable genetic resources for use in crop breeding, but their persistence is dependent on the presence of their pollinators, and at risk due to several anthropogenic pressures including climate change. To contribute to the safeguarding of this wild *Vanilla* gene pool, the present study aims at better understanding the effects of climate change on *Vanilla* species and their pollinators, and to identify potential spatial mismatches between both. Focusing on the Neotropical realm, we used MaxEnt species distribution models (SDMs) to predict potential changes in the range overlap between *Vanilla* and their pollinators by 2050 under the SSP2-4.5 and SSP3-7.0 climate change scenarios. We were able to compile enough occurrence records to generate SDMs for 11 Neotropical *Vanilla* CWRs, of which data on pollinator identity was available for four animal-pollinated species. Our models showed varying results among *Vanilla* species, with some predicted to undergo a net contraction (-1% to -53%) and others predicted to experience a net expansion (+11 to +140%), while the area of suitable habitat for all pollinators was predicted to decline (-7% to -71%). Our models predict a decline in range overlap between animal-pollinated *Vanilla* species and their pollinators under climate change, and this spatial mismatch was more pronounced for species reliant on a single known pollinator (-60% to -90%). Furthermore, the proportion of overlapping ranges located within protected areas is predicted to shrink for all species if no action is taken. Based on these findings, we propose priority areas for *in situ* and *ex situ* conservation to safeguard *Vanilla*’s genetic resources.

## 1 Introduction

Climate change is expected to cause substantial shifts in species’ geographic ranges, thereby altering the composition of species communities and disrupting interspecific interactions (Scheffers et al., 2016). The relationship between a plant and its pollinator(s) is an example of an ecological interaction that may be at risk due to differential responses of species to climate change, which may result in spatial mismatches between their future distributions (Gérard et al., 2020). Pollination contributes to species coexistence within plant communities, affects their geographic range, and drives evolutionary phenomena such as reproductive isolation or diversification rates between plant lineages (Phillips et al., 2020). The great majority (± 87.5%) of known flowering plants rely on animal vectors for cross-pollination (Ollerton, 2021), which are essential for shaping the genetic structure of populations of flowering plants by facilitating pollen (and gene) flow within and between populations. This process enables the spread of beneficial mutations that support adaptive responses to environmental changes (Conner and Hartl, 2004), so disruptions to this fundamental relationship could significantly reduce plant reproductive success and survival. Understanding the factors driving spatial and temporal changes in plant-pollinator networks is therefore critical for maintaining community structure and function and for developing efficient biodiversity conservation strategies (Burkle and Alarcón, 2011).

An important group of plants that is at risk due to climate change and other human-induced changes are crop wild relatives (CWRs) (Castañeda-Álvarez et al., 2016). CWRs are closely related to domesticated crop species and harbor a wealth of – often untapped – genetic diversity vital for crop improvement (Maxted et al., 2006; Vincent et al., 2019). Moreover, approximately 75% of plants used in food production depend, at least partially, on pollination by animal vectors, making pollinators essential to both natural and agricultural ecosystems (Van der Sluijs and Vaage, 2016). Understanding how climate change affects the range dynamics and ecological interactions of CWRs is therefore critical for protecting this wild gene pool and ensuring future food security.

An example of a crop with several wild relatives spread across the tropics is vanilla (*Vanilla* Mill., Orchidaceae Juss.), a globally valued spice and the most important orchid used in the food industry. Cultivated lineages of the commercial crop species *Vanilla planifolia* Andrews are, however, susceptible to biotic (e.g., pests, diseases) and abiotic (e.g., droughts, heat) stresses (Besse, 2004; Schlüter et al., 2007; Bory et al., 2008). Climate change is expected to aggravate their vulnerability to these stresses, leading to significant global yield declines (Bramel and Frey, 2021; Goettsch et al., 2021; Armenta-Montero et al., 2022; Karremans, 2024). Strengthening the resilience of vanilla cultivation systems will be essential to meet the growing demand for natural vanilla (Climate Bonds Initiative (CBI), 2023), with *Vanilla* CWRs playing a crucial role (Flanagan and Mosquera-Espinosa., 2016; Pérez-Silva et al., 2021, 2025; Bramel and Frey, 2021; de Oliveira et al., 2022; da Silva Oliveira et al., 2022; Watteyn et al., 2023a). *Vanilla* CWRs include wild populations of *V. planifolia*, as well as related species belonging to the same (*Vanilla* sect. *Xanata*) or to different sections as *V. planifolia* (*Vanilla* sect. *Tethya*, *Vanilla* subg. *Vanilla*). Many of these are considered as (critically) endangered by the IUCN Red List (Hernández-Fernández et al., 2020; Herrera-Cabrera et al., 2020; Wegier et al., 2020). Anthropogenic pressures such as climate change, habitat conversion, agricultural intensification, and illegal extraction from the wild are threatening the survival of remaining *Vanilla* CWR populations (Goettsch et al., 2021). Urgent action is therefore required to implement policies that support both *in situ* and *ex situ* conservation to safeguard these genetic resources (Bramel and Frey, 2021; Goettsch et al., 2021; Karremans, 2024).

Several *Vanilla* species, including *V. planifolia*, are self-compatible (Bory et al., 2010), explaining the success of hand-pollination in commercial plantations. However, in their natural habitat, most *Vanilla* species appear to be allogamous and rely on biotic vectors for sexual reproduction (Bory et al., 2010; Karremans, 2024). This dependence on pollinators corresponds with the broader pattern in orchids, where about 75% of the species require animal vectors for pollination (Ackerman et al., 2023). To date, effective pollinators of *Vanilla* species have been identified across several bee tribes, including Allodapini (Petersson, 2015; Gigant et al., 2014, 2016), Anthophorini (Gigant et al., 2014), Centridini (Nielsen & Ackerman unpublished; Pansarin et al., 2013), Euglossini (Ackerman, 1983; Lubinsky et al., 2006; Householder et al., 2010; Soto Arenas and Dressler, 2010; Pansarin et al., 2013; Anjos et al., 2017; Watteyn et al., 2022, 2023b), and Halictini (Chaipanich et al., 2020). Several previous studies cited stingless bees (Meliponini) as the suspected pollinators of *Vanilla*, but without clear evidence (e.g., Bouriquet 1946; Pijl and Dodson, 1996; Fouché and Jouve, 1999). A recent study of Karremans (2024) shows a stingless bee with pollen grains on its back while exiting a *V. planifolia* flower and suggested that these bees could remove pollinaria on occasion, but that it is unlikely that they are the main pollinators, given their small size. Pansarin and Ferreira (2022a) reported hummingbirds as pollinators of *Vanilla palmarum*, yet their statement lack evidence of pollen removal. The abovementioned pollinator groups interact with *Vanilla* species through various mechanisms, such as nectar rewards in the case of *Vanilla hartii* Rolfe (Watteyn et al., 2023b) or a food deceptive strategy in the case of *V. planifolia* (de Oliveira et al., 2022; Pemberton et al., 2023). Other species, such as *Vanilla pompona* Schiede, employ a dual mechanism with floral fragrances to attract pollinators and food deception to induce pollen removal and deposition (Watteyn et al., 2022; Pansarin, 2023). Plant species with such specialized pollinator interactions are expected to be more vulnerable to climate change-induced plant-pollinator decoupling than more generalist species (Gérard et al., 2020). As such, *Vanilla* species and their pollinators may be at risk of a spatial mismatch under changing climate conditions.

To support the conservation of *Vanilla* CWRs and their pollinator interactions, a critical first step is to identify those areas where they co-occur, and how these areas may change under projected climate change scenarios. Species distribution models (SDMs) provide a useful tool for this purpose, as they generate predictions of the distribution of suitable habitat, even for species with limited occurrence data, supporting targeted conservation actions (Guisan and Thuiller, 2005; Hirzel et al., 2006). Moreover, they can be used to predict spatial (mis)matches between species by overlaying single species distribution predictions or using joint SDMs. Previous studies on orchid conservation, for example, used SDMs to identify potential spatial mismatches in future distributions between orchids and their pollinators, information that can subsequently be integrated in land management policies (e.g., Tsiftsis and Djordjević, 2020; Kolanowska et al., 2021a, 2021b; 2023; Liu et al., 2024). As for *Vanilla*, however, existing SDM studies centered on current and future distribution patterns of the commercial crop species *V. planifolia* in Mexico (Hernández-Ruíz et al., 2016; Armenta-Montero et al., 2022; Maceda et al., 2023) and *Vanilla* CWR in Costa Rica (Watteyn et al., 2020). Rather than SDMs, Ellestad et al. (2021) applied a landscape-based approach to circumscribe the current geographical distribution of *V. planifolia* by accounting for the co-occurrence of pollinators and seed dispersers, as well as habitat quality and disturbance. No studies to date have modeled *Vanilla* species alongside their pollinators under predicted climate change scenarios, leaving a significant gap in understanding the spatial dynamics critical for their conservation.

The present study aims to evaluate the current overlap of suitable habitats between *Vanilla* CWRs and their pollinators, as well as to predict how this overlap might shift under future climate conditions. The focus is on tropical America, which harbors at least 63 of the 118 *Vanilla* species naturally found across the tropics (Karremans et al., 2020). Interestingly, this area also harbors all the so-called aromatic species (38 in total) belonging to the section *Xanata*, which are the species with most potential for use in crop breeding. We use MaxEnt SDMs to predict changes (contraction or expansion) in the range overlap between *Vanilla* and their pollinator species under the SSP2-4.5 and SSP3-7.0 climate change scenarios. The findings of this study can help to prioritize *in situ* conservation areas where both *Vanilla* species and their pollinators are predicted to continue to co-exist. Additionally, by identifying areas predicted to lose suitability, the results can be used to identify potential locations of *Vanilla* populations that may require *ex situ* conservation or assisted migration.

## 2 Materials and methods

### 2.1 Species distribution modeling

#### 2.1.1 Occurrence data

Georeferenced presence data of all currently known Neotropical *Vanilla* species (n = 63) (**Supplementary Table S1**) was compiled from several sources, including Karremans et al. (2020) and Watteyn et al. (2020), among others (see **Supplementary Table S2** for a complete overview), and complemented with data recently collected by our research group (2023-2024) as part of an ongoing genetic study of *Vanilla* populations (Watteyn et al., in prep.). More specifically, we compiled presence data within the geographical extent of (sub)tropical America considering the currently known distribution of Neotropical *Vanilla* species (-118.37°W, -28.85°E; -33.75°S, 32.72°N). We cleaned the presence data using the R package CoordinateCleaner (Zizka et al., 2019) and removed (i) records located in the ocean, GBIF headquarters, urban areas or biodiversity institutions (e.g., museums, botanical gardens, universities), and (ii) records with outlier, zero, rounded or invalid coordinates, and identical latitude/longitude. We also removed records older than 1950 or with missing collection dates. After spatial filtering (see section 2.1.3.), only *Vanilla* species with ≥ 30 occurrence points were retained, as the number of presence points greatly affects model accuracy (Wisz et al., 2008). This resulted in a total of 11 *Vanilla* species that could be modeled, including 7 animal-pollinated and 4 autogamous species (**Table 1**). Information about the pollinators of the animal-pollinated *Vanilla* species was derived from recent studies, resulting in 11 potential pollinators described in the literature, of which seven are supported by robust observations of pollen removal and identifications of the pollinators at species level (**Table 1**). Georeferenced presence data of these seven pollinator species was compiled from GBIF and literature (**Supplementary Table S2**). The data were cleaned with the same procedure as for the *Vanilla* species, to obtain a final dataset comprising seven pollinator species with sufficient occurrence data (≥ 30 points). All modeled species belonged to the bee tribe Euglossini, including four *Euglossa* and three *Eulaema* species.

**Table 1:** Set of Neotropical *Vanilla* species (N = 11) with enough presence data to build accurate models, along with the identified pollination mechanism and corresponding pollinator species in case of animal-driven allogamy.

	*Vanilla* species	Pollination strategy	Pollinator species	References
*Vanilla* sect. *Xanata*	*Vanilla chamissonis* Klotsch	Autogamous	n.a.	Reis, 2000; Gigant et al., 2011
	***Vanilla hartii* Rolfe**	Animal-pollinated | Nectar-rewarding	***Euglossa cybelia*** ***Euglossa tridentata***	Watteyn et al., 2023b; Watteyn et al., 2023b
	*Vanilla odorata* C. Presl.	Animal-pollinated | Food-deceptive	*Euglossa* sp.^b, d^	Soto Arenas and Dressler, 2010; Watteyn et al. unpubl.
	*Vanilla palmarum* Lindl	Autogamous	n.a.	Householder et al., 2010; Soto Arenas and Cribb, 2013
	*Vanilla phaeantha* Rchb.f.**^a^**	Animal-pollinated | Food-deceptive	*Eulaema* sp.^b^	Anjos et al., 2017
	***Vanilla planifolia*** **Andrews**	Animal-pollinated | Food-deceptive	*Euglossa viridissima* ^b^ ***Euglossa dilemma* ^c^** *Trigona* sp.^b^	Soto Arenas and Dressler, 2010; Pemberton et al., 2023; Karremans, 2024
	***Vanilla pompona* Schiede**	Animal-pollinated | Dual mechanism	***Eulaema cingulata*** ***Eulaema meriana*** ***Eulaema nigrita***	Watteyn et al., 2022; Lubinsky et al., 2006; Householder et al., 2010; Ackerman, 1983
	***Vanilla trigonocarpa*** **Hoehne**	Animal-pollinated | Food-deceptive	***Euglossa asarophora*** *Eulaema meriana* ^d^	Soto Arenas and Dressler, 2010 Karremans et al. unpubl.
*Vanilla* subg. *Vanilla*	*Vanilla bicolor* Lindl.	Autogamous	n.a.	Householder et al., 2010; Van Dam et al., 2010
	*Vanilla inodora* Schiede	Autogamous Animal-pollinated	n.a. no information	Soto Arenas and Dressler, 2010; Soto Arenas and Dressler, 2010
	*Vanilla mexicana* Mill.	Autogamous	n.a.	Gigant et al., 2016

^a^ *Vanilla phaeantha* Rchb.f. is a synonym to *Vanilla bahiana* Hoehne (Karremans et al., 2020). ^b^No observation of pollen removal and/or no identification at species level, so data not accurate enough for our study. ^c^Study performed outside native distribution range of *V. planifolia* (Florida), but data sufficiently accurate for our study (*Euglossa dilemma* may be a pollinator within *V. planifolia*’s native range). ^d^Reference to unpublished publication, so data not used in our study.

#### 2.1.2 Predictor variables

As predictor variables, we used the bioclimatic variables from the WorldClim database, with a spatial resolution of 30 arcsec (ca. 0.9 km at the equator), both for the near-current historical baseline (1970-2000) and future (2041-2070) climate conditions (Fick and Hijmans, 2017). Following Booth (2022), the variables bio8, bio9, bio18 and bio19 were removed due to known spatial artefacts. No further variable selection was carried out, as Maxent models can handle multicollinearity (Feng et al., 2019). For the *Vanilla* SDMs, we also included eight soil variables with a spatial resolution of 250 m (SoilGrids) from the International Soil Reference and Information Center (ISRIC, Hengl et al., 2017) and 4 topographic variables with a spatial resolution of 30 m from the ASTER Global Digital Elevation Model v3 (DEM, Abrams et al., 2022), both resampled to a resolution of 30 arcsec to match the resolution of the bioclimatic variables. An overview of the predictor variables can be found in **Supplementary File 1** (**Supplementary Table S3**). The pollinator SDMs only included climatic variables, as previous studies have shown that bee distribution ranges are mainly driven by climate, and other variables do not significantly improve the models (Silva et al., 2014; Nemésio et al., 2016).

We selected five general circulation models (GCMs) from the sixth Coupled Model Intercomparison Project (CMIP6) (Eyring et al., 2016) with the highest combined weight of performance (i.e. ability to predict past climate conditions) and independence according to Brunner et al. (2020) that are available through the WorldClim database: ACCESS-CM2, GISS-E2-1-G, INM-CM5-0, MIROC6, MPI-ESM1-2-HR. For each of these GCMs, we focus on two climate change scenarios: the Shared Socioeconomic Pathways SSP2-4.5 and SSP3-7.0 (Riahi et al., 2017). These SSPs are projections in terms of international policies towards environmental sustainability and GHG emission reduction. SSP2-4.5 (“middle of the road”) assumes that nations will work toward but make slow progress in achieving sustainability in development goals, while SSP3-7.0 (“rocky road” – regional rivalry) is a more pessimistic scenario, with greater regional conflicts and less global cooperation to mitigate climate change. We choose these two SSP scenarios as we aimed to include scenarios which may best reflect reality, considering a more optimistic and pessimistic vision, respectively. The other scenarios reflect very optimistic or pessimistic views on the future. For example, SSP1-1.9 and SSP1-2.6 envision a world where ambitious mitigation efforts lead to significant GHG reductions, reaching net-zero emissions by 2050 or 2070, respectively, while the very pessimistic SSP5-8.5 scenario envisions a world where emissions continue to grow at very high rate, which is unlikely (Huard et al., 2022).

#### 2.1.3 Species distribution modeling

We used the maximum entropy algorithm (MaxEnt version 3.4.3) (Phillips et al., 2006; Elith et al., 2011) to model the distribution of *Vanilla* species and their corresponding pollinators under current and future climate conditions. MaxEnt has become a popular tool for predicting species distributions, as it can cope well with sparse, irregularly sampled data and minor location errors (Graham et al., 2008). MaxEnt is a niche modeling algorithm based on the maximum entropy theory (Phillips et al., 2006, 2017). It is a presence-only algorithm that compares presence locations to all the environments that are available in the study region, i.e. the ‘background’.

To reduce the effects of spatial bias on model calibration, we applied the target background approach, which involves the selection of background records from grid cells with presence data of species that belong to a similar group as the target species, under the assumption that these locations reflect a similar bias as the sampling bias of the target species (Phillips et al., 2009). In our case, the target group for the *Vanilla* SDMs consisted of all hemi-epiphyte and liana species growing in the Neotropics (tropicos.org), while the target group for the pollinator SDMs consisted of all bee species (Apidae) found in the Neotropics (Dorey et al., 2023). Presence data of the target group species were compiled from online databases and the literature (**Supplementary Table S2**) and cleaned using the same method as explained in section 2.1.1. To further reduce the effects of spatially biased presence points on model calibration, we thinned the presence points using the R package spThinR (Aiello-Lammens et al., 2015), using a thinning distance of 10 km.

The MaxEnt models were implemented and optimized using the R package ENMeval v2 (Kass et al., 2021). For each species, a total of 15 model parametrizations were evaluated by using multiple combinations of five feature classes (L, LQ, H, LQH, LQHP, where L = linear, Q = quadratic, H = Hinge, P = Product) and three regularization multiplier (RM) values (1, 3, 5). To evaluate the models, we performed a spatial block cross-validation using the R package blockCV (Valavi et al., 2019), in which presence and background data were divided into 100 km wide squared blocks arranged in eight cross-validation folds. To obtain the best model among the 15 models, we first chose the four models with the highest Area Under the receiver-operating characteristic Curve (AUC), and then selected the model with the smallest difference between training and testing AUC, which is a measure for overfitting (i.e. among the four models with highest AUC we selected the model with least overfitting). The use of AUC has been criticized, mainly because AUC values are easily inflated by increasing the geographical (i.e. environmental) extent in which background points are selected (Lobo et al., 2010). To avoid this, we only selected background points within a convex hull around the presence records, extended by a buffer of 20% of the longest distance between presence records. Projections were made for the entire geographical extent of (sub)tropical America (see 2.1.1) but further analysis and interpretation was restricted to the area encompassed by the convex hulls, to minimize extrapolation to conditions under which the models were not trained. Models with AUC values greater than 0.7 (i.e., acceptable accuracy; Raes & ter Steege, 2007) were selected for further analysis.

The final models were used to predict habitat suitability under both current and future conditions. Suitability maps were converted to presence-absence maps using the threshold at which the sum of the sensitivity (true positive rate) and specificity (true negative rate) was highest (Liu et al., 2005; Jimenez-Valverde and Lobo., 2007). From the five GCM binary outputs for each SSP, we used a majority vote rule to predict suitability to generate a single output for future projections. We then calculated changes (km^2^) in habitat suitability (contraction, expansion, no change) between current and future distributions for all modeled species separately. All analyses were carried out in R v4.3.3 (R Core Team, 2025) and the final maps were visualized using QGIS v3.40.

### 2.2 *Vanilla*-pollinator range overlap and identification of priority conservation areas

QGIS v3.40 was used to visualize the overlap in distribution range (hereafter “range overlap”) between the animal-pollinated *Vanilla* species and its known pollinator(s) and to assess changes in this overlap under the two SSP scenarios by 2050. We calculated the area of range overlap between each *Vanilla* species and its pollinator(s) under current and future climate conditions. For *Vanilla* species with more than one known pollinator, we summed the presence maps of their individually modeled pollinator species before overlaying them with the presence map of the corresponding *Vanilla* species. Presence-absence maps displaying habitat suitability for *Vanilla* species and their pollinator(s) were then used to identify areas suitable for *in situ* conservation and to highlight *Vanilla* populations that may require *ex situ* conservation or assisted migration. Using the World Database on Protected Areas (WDPA) map (UNEP-WCMC & IUCN, 2024), we assigned high-priority *in situ* conservation areas where *Vanilla* species and their pollinator(s) are predicted to continue to coexist under future climate scenarios. We distinguish between areas already under protection, and priority conservation areas that need to be established (i.e. no protection status at present). Furthermore, we identified populations located in areas that are expected to become unsuitable by 2050 and may need *ex situ* conservation (e.g., in botanical gardens) or assisted migration (e.g., relocation of these populations to areas expected to remain or become suitable).

## 3 Results

### 3.1 Climate change effects on the distribution of *Vanilla* and its pollinators

The *Vanilla* and pollinator models showed a high level of predictive accuracy (average AUC = 0.84 ± 0.07 SD and average AUC = 0.81 ± 0.09 SD, respectively) (**Supplementary Table S4**). Based on the permutation importance, we found that the most important variables predicting the current distribution of the modeled *Vanilla* species were related to climate rather than soil variables (**Supplementary Tables S4**, **S5**). Specifically, the distribution of five species (*V. bicolor*, *V. hartii*, *V. mexicana*, *V. phaeantha*, *V. trigonocarpa*) was mainly predicted by precipitation variables, including annual precipitation (bio12), precipitation of driest month (bio14), and precipitation seasonality (bio15). The habitat suitability of the other six species (*V. chamissonis*, *V. inodora*, *V. odorata*, *V. palmarum*, *V. planifolia*, and *V. pompona*) was primarily predicted by temperature variables such as temperature seasonality (bio4), minimum temperature of coldest month (bio6), and temperature annual range (bio7). Moreover, we found that the distributions of *V. bicolor*, *V. phaeantha*, and *V. pompona* is also predicted by soil pH. The remaining climate, soil and topography variables seem to be less important in predicting *Vanilla* species distributions. The distribution of the pollinators is mainly predicted by temperature variables (**Supplementary Tables S4**, **S6**), including annual mean temperature (bio1 - *Euglossa tridentata*, *Eulaema meriana*), mean diurnal range (bio2 - *Euglossa cybelia*), mean temperature of coldest month (bio6 - *Eulaema cingulata*), and mean temperature of coldest quarter (bio11 - *Euglossa asarophora*, *E. dilemma*, *Eulaema nigrita*).

**Figure 1:**
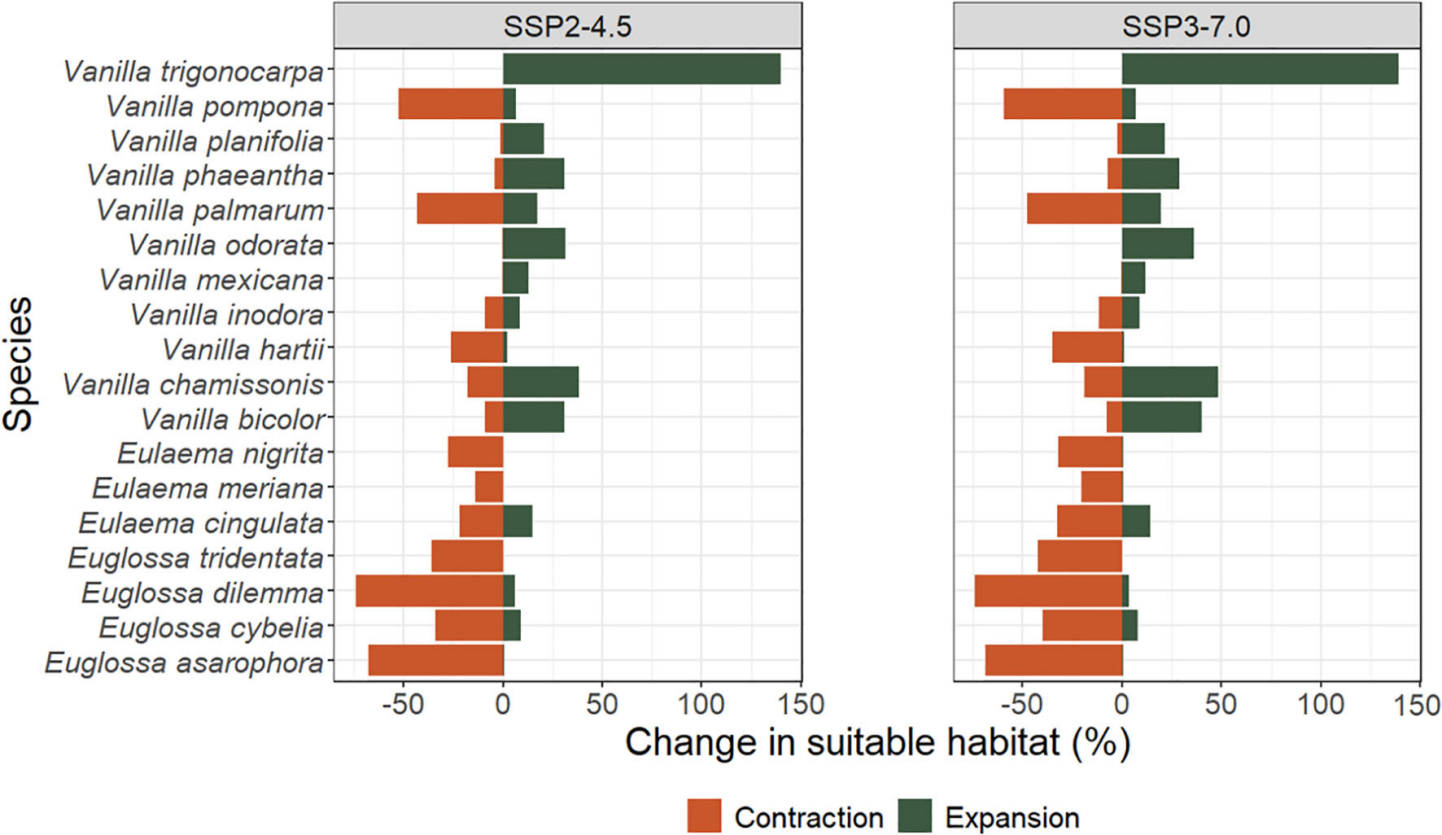
Predicted changes in suitable habitat by 2050 under the “middle of the road” (SSP2-4.5) and “regional rivalry” (SSP3-7.0) scenarios for the 11 modeled *Vanilla* species and seven pollinator species. Calculations were made considering the area encompassed by the convex hulls around the presence points of the modeled species (see 2.1.3).

**Figure 1** shows the changes in habitat suitability of *Vanilla* and pollinator species predicted under both scenarios (SSP2-4.5 and SSP3-7.0) for the year 2050 relative to the near-current historical baseline (1970-2000), to which we will refer to as ‘present’ for simplicity. In the SSP2-4.5 scenario, the habitat suitability of four *Vanilla* species (*V. hartii*, *V. inodora*, *V. palmarum*, *V. pompona*) is predicted to decrease, with net changes in suitable area ranging from -1% to -46%. For the other seven species (*V. bicolor*, *V. chamissonis*, *V. mexicana*, *V. odorata*, *V. phaeantha*, *V. planifolia*, *V. trigonocarpa*), our models predicted an increase in habitat suitability, with net changes ranging from +12% to +140%. A similar trend is predicted under the SSP3-7.0 scenario, with a decrease (net change ranging from -3% to -53%) or increase (net change ranging from +11% to +139%) in habitat suitability for the same species.

The habitat suitability of all modeled pollinator species is predicted to decline, with slightly higher negative net changes under the SSP3-7.0 compared to the SSP2-4.5 scenario (**Figure 1**). The greatest reduction is predicted for the *Euglossa* species, with net changes ranging from -24.6% to -68.2% under the SSP2-4.5 scenario and -31.7% to -70.7% under the SSP3-7.0 scenario. The predicted decrease in habitat suitability for the three *Eulaema* species was less compared to the other pollinator species, with net changes ranging from -6.9% to -27.4% under the SSP2-4.5 scenario and -18.5% and -31.6% under the SSP3-7.0 scenario.

*Vanilla* and pollinator presence-absence maps for current climate conditions as well as the maps demonstrating the predicted future changes can be found in **Supplementary File 1** (**Supplementary Figures S1**, **S2**), together with an overview of the predicted changes in suitable habitat (km^2^) and net change (%) for both *Vanilla* and pollinator species (**Supplementary Table S7**).

### 3.2 Climate change-induced shifts in *Vanilla*-pollinator range overlap

**Figure 2:**
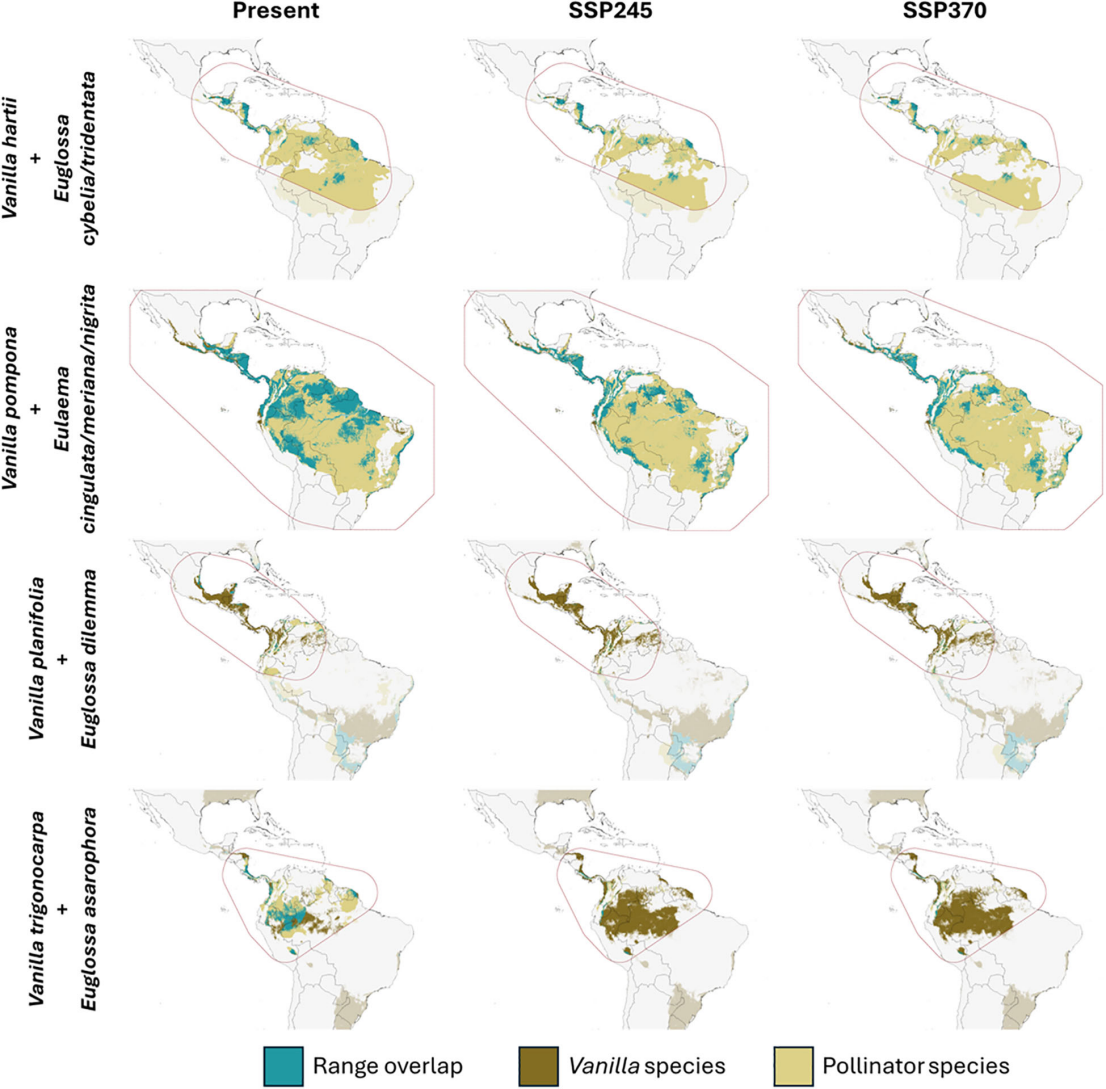
Maps showing the range overlap between animal-pollinated *Vanilla* species and their corresponding pollinator(s) under present climate conditions (left) and predicted climate change scenarios SSP2-4.5 (middle) and SSP3-7.0 (right) for 2050. Projections were made for the entire geographical extent of (sub)tropical America but interpretation was restricted to the area encompassed by the convex hulls (red dotted line) under the assumption that models are extrapolated more outside these hulls. Areas outside the hulls were given a lighter color.

**Table 2** shows the predicted climate change-induced shifts in *Vanilla*-pollinator range overlap for the animal-pollinated *Vanilla* species for which data on pollinators was available (i.e. four of the in total 11 modeled *Vanilla* species): (i) *V. hartii* and pollinators *Euglossa cybelia* and *E. tridentata*, (ii) *V. planifolia* and pollinator *Euglossa dilemma*, (note: observations of pollen removal made by Pemberton et al. (2023) took place outside the native distribution range of *V. planifolia* (Florida) but *Euglossa dilemma* has been recorded within *V. planifolia*’s native range hence may be considered as a pollinator), (iii) *V. pompona* and pollinators *Eulaema cingulata*, *V. meriana*, and *E. nigrita*, and (iv) *V. trigonocarpa* and pollinator *Euglossa asarophora*. Overall, our models predict a decrease in range overlap by 2050 (**Figure 2**). This predicted spatial mismatch is slightly larger in the SSP3-7.0 scenario for *V. hartii*, *V. pompona*, and *V. trigonocarpa*, while it is very similar in both the SSP2-4.5 and SSP3-7.0 scenarios for *V. planifolia* and *V. trigonocarpa*. The largest spatial mismatch is predicted for *V. trigonocarpa*, with a decline in plant-pollinator range overlap of about 90% relative to the present situation, followed by *V. planifolia*, *V. pompona*, and *V. hartii*.

**Table 2:** Area of range overlap (km^2^) between *Vanilla* species and their pollinator(s) under present and future climate conditions, and the net change in range overlap between present and future climate conditions (%).

*Vanilla* species	Pollinator species	Scenario	Range overlap (km^2^)	Net change in range overlap by 2050 (%)
*Vanilla hartii*	*Euglossa cybelia*	Present	314,622	
		SSP2-4.5	221,659	- 29.6
		SSP3-7.0	165,776	- 47.3
	*Euglossa tridentata*	Present	576,333	
		SSP2-4.5	423,937	- 26.4
		SSP3-7.0	342,925	- 40.5
	Both pollinators	Present	581,827	
		SSP2-4.5	430,601	- 26.0
		SSP3-7.0	349,032	- 40.0
*Vanilla planifolia*	*Euglossa dilemma*	Present	123,015	
		SSP2-4.5	44,705	- 63.6
		SSP3-7.0	48,700	- 60.4
*Vanilla pompona*	*Eulaema cingulata*	Present	3,729,349	
		SSP2-4.5	2,128,650	- 42.9
		SSP3-7.0	1,793,170	- 51.9
	*Eulaema meriana*	Present	3,690,869	
		SSP2-4.5	1,440,330	- 61.0
		SSP3-7.0	1,130,422	- 69.4
	*Eulaema nigrita*	Present	3,602,796	
		SSP2-4.5	1,634,702	- 54.6
		SSP3-7.0	1,381,341	- 61.7
	All three pollinators	Present	4,387,376	
		SSP2-4.5	2,281,764	- 48.0
		SSP3-7.0	1,955,612	55.4
*Vanilla trigonocarpa*	*Euglossa asarophora*	Present	619,237	
		SSP2-4.5	67,845	- 89.0
		SSP3-7.0	59,830	- 90.2

**Figure 3:**
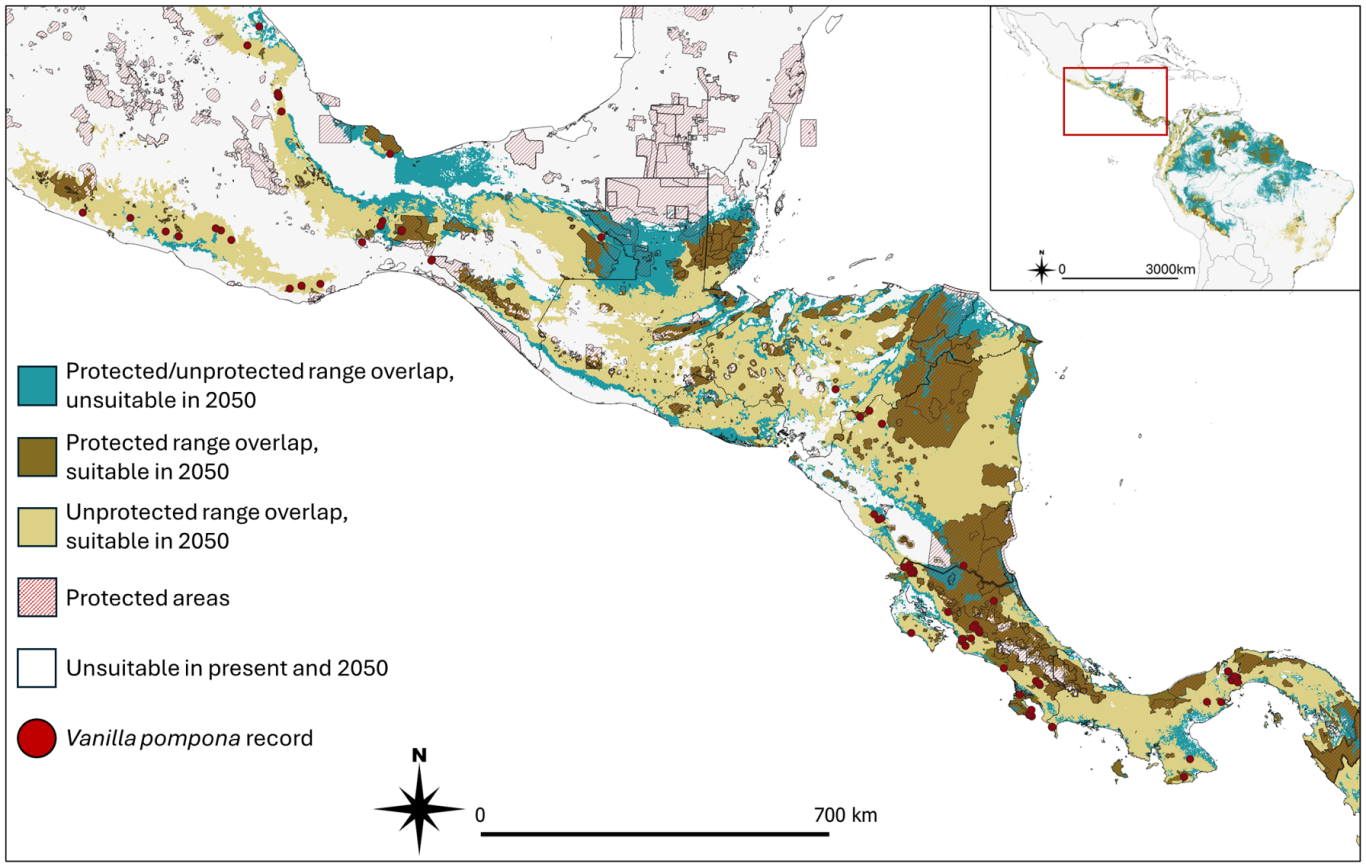
Map indicating currently protected areas where the range of a *Vanilla* species and its pollinator(s) was predicted to overlap in 2050 (dark brown), currently unprotected areas with a predicted range overlap between a *Vanilla* species and its pollinator(s) in 2050, which could be prioritized new conservation areas (light brown), and areas that harbor populations that may need *ex situ* conservation or assisted migration as they are found in areas that are predicted to become unsuitable by 2050 (blue). We used the species *V. pompona* and the SSP2-4.5 scenario as an example.

**Table 3** gives an overview of the proportion of protected suitable areas shared between *Vanilla* species and their pollinators under present and future climate conditions. For example, of the total amount of area predicted to be suitable for both *V. pompona* and its pollinators (i.e., range overlap) under present climate conditions, about 42% is currently protected. By the year 2050, the proportion of protected shared suitable area is expected to decrease to about 21% (SSP2-4.5) and 17% (SSP3-7.0). *Vanilla* species with multiple known pollinators (*V. hartii* and *V. pompona*) have a higher proportion of protected shared habitat compared to those with only a single known pollinator (*V. planifolia* and *V. trigonocarpa*). All *Vanilla* species show a decreasing trend of protected *Vanilla*-pollinator shared area by 2050 if no actions are taken. **Figure 3** shows a map indicating priority conservation areas, using *V. pompona* as an example. The same maps for the other *Vanilla* species are available in the **Supplementary File 1** (**Supplementary Figure S3**). These maps show (i) currently protected areas where the range of a *Vanilla* species and its pollinator(s) overlap, (ii) currently unprotected areas with range overlap between a *Vanilla* species and its pollinator(s), which could be prioritized new conservation areas, and (iii) areas that harbor populations that may need *ex situ* conservation or assisted migration, as they are predicted to become unsuitable in the future.

**Table 3:** Overview of the proportion of range overlap between a *Vanilla* species and its known pollinator(s) located within protected areas, and this under model predictions for present and future (SSP2-4.5 and SSP3-7.0) climate conditions.

*Vanilla* species	Scenario	Proportion of shared suitable area within protected areas (%)
***Vanilla hartii*** Pollinators: *Euglossa cybelia, E. tridentata*	Present	55.6
	SSP2-4.5	39.6
	SSP3-7.0	31.3
***Vanilla planifolia*** Pollinators: *Euglossa dilemma*	Present	31.0
	SSP2-4.5	14.7
	SSP3-7.0	15.9
***Vanilla pompona*** Pollinators: *Eulaema cingulata, E. meriana, E. nigrita*	Present	41.9
	SSP2-4.5	21.0
	SSP3-7.0	16.8
***Vanilla trigonocarpa*** Pollinators: *Euglossa asarophora*	Present	42.5
	SSP2-4.5	4.5
	SSP3-7.0	4.0

## 4 Discussion

Focusing on the crop wild relatives (CWRs) of the high-value cash crop vanilla, we generated SDMs for 11 Neotropical CWRs, of which data on pollinator identity was available for four animal-pollinated species, including the commercially cultivated *V. planifolia*. The models showed varying results among *Vanilla* species, with some predicted to undergo a net contraction and others predicted to experience a net expansion. However, all four animal-pollinated species were predicted to experience a decline in range overlap with their pollinators. This spatial mismatch was even more pronounced for *Vanilla* species reliant on a single known pollinator. At present, the proportion of shared suitable habitats located within protected areas varies among *Vanilla* species, but strong declines are expected for all species by 2050 in case no action is taken. Our spatially explicit results can be used to guide *in situ* and *ex situ* conservation strategies.

### 4.1 Varying effects of climate change on the distribution of *Vanilla* and its pollinators

Climate change is expected to cause a decline in the area of suitable habitat of the modeled *Vanilla* species, as orchids are known to have higher extinction rates, tend to inhabit narrower habitats, and are more susceptible to disturbances than many other plants (Gravendeel et al., 2004; Cozzolino and Widmer, 2005; Swarts and Dixon, 2009; Shrestha et al., 2021). Also, the only *Vanilla* SDM study (Armenta-Montero et al., 2022) comparing present and future habitat suitability of *V. planifolia* predicted a progressive reduction in both cultivated and natural distribution areas. Conversely, our models forecasted varying results among *Vanilla* species, with a net expansion in area of suitable habitat predicted for some species and a net contraction for others. These findings align with previous studies showing varying responses of orchids to climate change, even among closely related species currently occupying similar habitats (e.g., Evans et al., 2020; Kolanowska et al., 2020; Smallwood and Trapnell, 2022; Qiu et al., 2023; Liu et al., 2024).

The area with suitable habitat of four species is predicted to decrease by 2050, with greater declines in the SSP3-7.0 compared to the SSP2-4.5 scenario. The higher vulnerability of these species to climate change may be due to the prevalence of species-specific plant traits and adaptations to specific climate conditions leading to narrower environmental niches, amongst others. For example, *V. inodora* only inhabits cloud forests and lowland sites with more than 2500 mm of rainfall (Soto Arenas and Dressler, 2010), while *V. palmarum* mainly occurs in hot and semi-arid regions with a long dry season (e.g., Caatinga and Atlantic Forest of Brazil). Moreover, *V. palmarum* has a phorophyte specificity with certain palm species (Householder et al., 2010; Barberena et al., 2019 and references herein), and considering this phorophyte dependency in future models might result in even stronger declines. As stated before by Aitken et al. (2007) and shown in previous studies (e.g., Thuiller et al., 2005; Kolanowska, 2023; Fan and Luo, 2024; Cho et al., 2024; Wysocki et al., 2024), these kind of specificities can greatly limit a plant’s distribution under changing environmental conditions.

The models predicted an increase in area with suitable habitat for the remaining seven *Vanilla* species, meaning that climate conditions for these species may become more favorable by 2050. For example, *V. odorata* has a large distribution and naturally grows in a range of bioclimatic regions (Jiménez et al., 2017). This wide niche breadth possibly leads to a higher tolerance to changing environmental conditions, as previously observed in species with wider niche breadths (e.g., Carrillo-Angeles et al., 2016; Evans et al., 2020). *V. phaeantha* seems to be more common in the drier lowland tropical rainforests (Soto Arenas and Dressler, 2010; Karremans et al., 2020). Future changes in precipitation will vary regionally, with some areas projected to become hotter and drier, especially in South America (Castellanos et al., 2022; Feron et al., 2024), hence driving the expansion of xerophytic species. Interestingly, our models predicted an increase in suitable habitat for *V. planifolia*. Previous research forecasted a progressive reduction in suitable area for this species in Mexico (Armenta-Montero et al., 2022). However, this study only used occurrence data from Mexico, which may lead to an overestimation of climate change impacts as a consequence of only covering a part of the species’ niche (Barbet-Massin et al., 2010). Our dataset included *V. planifolia* occurrence records across its entire native distribution range (Mexico to Colombia, Karremans et al., 2020).

The pollinator models predicted a decrease in suitable habitat, with greater declines expected for the smaller *Euglossa* bees compared to the larger *Eulaema* bees. Insect pollinators face worldwide declines due to climate and land use change, with species emerging earlier, phenological mismatching with floral resources, or changing range distributions (Whipple and Bowser, 2023). Most SDM studies of bees – generally seen as the most important plant pollinator group (Ollerton, 2021) – focused on the widespread bee genera *Bombus* and *Apis*, and forecasted contractions in distribution ranges, except for common species with larger niche breadths and dispersal capabilities (e.g., Casey et al., 2015; Kerr et al., 2015; Rasmont et al., 2015; Jacobson et al., 2018). Studies on other bee genera are scarce and have led to varying results. In the Neotropical realm, for example, research on orchid bees took primarily place in Brazil, with several species predicted to become more restricted under climate change (e.g., Giannini et al., 2012, 2013, 2020; Faleiro et al., 2018), while the suitable habitat of other species has been predicted to expand (e.g., Silva et al., 2015; Nemésio et al., 2016; Teixeira et al., 2018). Overall, however, a decrease in abundance, distribution, and diversity is expected for most orchid bees (Faleiro et al., 2018), and these changes are likely to disrupt plant-pollinator interactions, such as the ones between *Vanilla* species and their known Euglossini pollinators.

### 4.2 Climate-induced reductions in *Vanilla*-pollinator range overlap

Our models predicted varying responses for the modeled *Vanilla* species, with some species experiencing a contraction and others an expansion in the area of suitable habitat. However, a decrease in suitable habitat was predicted for all modeled pollinators, leading to strong reductions in range overlap between the animal-pollinated *Vanilla* species and their pollinators (**Table 3**). Pronounced declines were predicted for *V. planifolia* and *V. trigonocarpa*, species dependent on a single pollinator species (or at least with only one pollinator species known so far), as the area in suitable habitat of their pollinators (*Euglossa dilemma* and *E. asarophora*, respectively) within the distribution range of the corresponding *Vanilla* species is already limited at present. So despite the predicted increase in suitable habitat for the *Vanilla* species, their pollinator-dependency might imperil the remaining populations of these species.

The importance of assessing the distributions of both a plant and its pollinator(s) to predict the potential effects of a changing climate on a plant’s future distribution has been repeatedly recognized (e.g., Araújo and Luoto, 2007; Van der Putten et al., 2010; Engelhardt et al., 2020), especially for orchids given their specialized interactions with pollinators (e.g., McCormick and Jacquemyn, 2014; Robbirt et al., 2014; Ackerman et al., 2023). Tsiftsis and Djordjević (2020), for example, predicted a stronger decrease in suitable habitat for *Ophrys* species in models that integrated pollinator interactions compared to the ones without. Kolanowska et al. (2021a; 2021b; 2023). observed similar trends in other orchids (e.g., *Leporella*, *Limodorum*, *Traunsteinera*). Specifically, they predicted an expansion of the orchid’s geographical ranges under climate change, but due to the negative effects of climate change on their pollinators, their range overlap was predicted to decrease. These studies demonstrate a clear trend of plant-pollinator decoupling under climate change, affecting the distribution and genetic structure of corresponding species, and potentially leading to increased isolation (Karremans, 2024). In accordance with abovementioned studies, we highlight the importance of accounting for the highly specialized relationships between orchids and their pollinators to obtain more accurate insights into potential distributional changes under changing environmental conditions. Considering the observed plant-pollinator decoupling, the future may look brighter for autogamous species such as *V. bicolor*, *V. chamissonis* and *V. mexicana*, for which our models predicted increases in habitat suitability.

Pollinator specificity is common in the orchid family, with a median number of only one pollinator species, especially for species employing some means of deceit (Scopece et al., 2010; Ackerman et al., 2023). It is, however, possible that some of the animal-pollinated *Vanilla* species modeled in our study have more pollinators than the ones we identified based on the limited available literature, which could lead to higher functional redundancy and thus more resilient plant-pollinator networks. Ellestad et al. (2021), for example, using a landscape-based approach rather than SDMs, considered all *Euglossa* and *Eulaema* species as potential pollinators to determine the present distribution of *V. planifolia*, and predicted a larger potential distribution of this species when including the abovementioned Euglossini. Yet, their results must be interpreted with caution, as previous studies (e.g., Watteyn et al., 2022, 2023b) demonstrated the need for a morphological fit between vanilla flowers and bees for pollen removal to occur, restricting effective pollinator species to the ones showing a perfect fit with specific flower traits. This morphological fit could be used to select potential effective pollinators to be considered in future SDMs.

The existing knowledge gap in *Vanilla* pollination research clearly limits the current possibilities of SDMs, and thereby also the conservation efforts that can be informed by such modeling. Moreover, limited occurrence data further restricts the assessment of climate change effects on *Vanilla*-pollinator range overlap, as only 11 of the in total 63 Neotropical *Vanilla* species had enough occurrence records to model them, of which only four species are known to be animal-pollinated. Taking collaborative action to improve our knowledge on basic biological and ecological aspects is urgently needed to overcome these challenges (Karremans, 2023, 2024). This also includes data on other biotic interactions such as the ones between orchids and their seed dispersers, as well as microbial leaf litter and soil communities and mycorrhizae. Recent studies focusing on animal-mediated seed dispersal in *Vanilla* identified a wide range of seed dispersers of several *Vanilla* species, including bees (euglossine and stingless bees) and mammals (rodents, marsupials) (Karremans et al., 2023b, 2023a, Pansarin and Suetsugu, 2022b; Pansarin, 2024, 2025), providing the necessary information to assess potential future limitations in *Vanilla* distributions due to spatial mismatches with both pollinators and seed dispersers. In addition, *Vanilla* species also seem to depend on specific micro-organisms to ensure seed germination *in situ* (i.e., symbiotic germination) (e.g. Porras-Alfaro and Bayman, 2007; Alomia et al., 2017; Wong et al., 2024). A large knowledge gap still exists regarding this topic and future work untangling these symbiotic relationships would contribute to develop more comprehensive *Vanilla* conservation strategies. Finally, additional information on the effects of climate change on, for example, pollen germination and viability, and pollinator foraging, reproduction and emergence could further enhance our understanding of how *Vanilla* species could keep pace with global warming predictions.

### 4.3 Priority conservation areas for *Vanilla* and its pollinators

The loss of a subset of functionally important pollinator species can have a disproportionate impact on plant-pollinator networks (Leitão et al., 2016), and great concern exists about the possible disruptive effects of land use and climate change on the relationships between orchids and their complex ecological interactions (Karremans, 2023). Our models are a first step to indicate range overlap between a *Vanilla* species and its pollinator(s), and to assess if these areas are currently under protection or not. The map created for *V. pompona* (**Figure 3**) (maps for other species can be found in the **Supplementary Figure S3**) specifies *in situ* conservation areas as well as areas potentially holding populations that may need *ex situ* conservation or assisted migration. Specifically, areas with suitable habitat for *V. pompona* and its pollinators (i.e., range overlap) are areas that need conservation prioritization (especially areas currently holding known *V. pompona* populations). Yet, the priority further depends on the location, with areas of range overlap inside protected areas (less concern as already protected) or outside of protected areas (priority areas for establishing new conservation areas) protected areas. Areas predicted to become unsuitable in the future but currently holding *V. pompona* populations may require *ex situ* conservation (i.e. in botanical gardens or seed banks) or assisted migration to green areas (i.e. existing protected areas overlapping with area suitable for *Vanilla* and pollinator species in 2050). We need to recognize, however, that these outcomes may shift when more information would become available on *Vanilla* pollinators.

### 4.4 Concluding remarks

Although an increase in habitat suitability may be expected for some *Vanilla* species based on changes in climatic conditions, there are several other factors besides climate (e.g., habitat destruction and degradation, ecological interactions) that are limiting the geographical extent of a species. Our study showed that climate change may lead to reduced overlap in suitable habitats for *Vanilla* species and their pollinators, thereby causing plant-pollinator decoupling and possibly affecting the survival of *Vanilla* populations. Moreover, the predicted proportion of shared future habitat is relatively limited. The spatially explicit recommendations made using the modeled distribution ranges and range overlap are a first step to develop comprehensive conservation strategies for *Vanilla* and its pollinators across the Neotropics. Future studies could integrate detailed information on species population biology and life-history dynamics, behavior plasticity and genetic adaptation as well as land management and restoration strategies to further refine conservation priorities.

## Data Availability

The datasets presented in this study can be found in online repositories. The names of the repository/repositories and accession number(s) can be found below: Dryad repository.
