# Supplementary material for: Wild Vanilla and pollinators at risk of spatial mismatch in a changing climate
Source: Front Plant Sci. 2025 Jul 3;16:1585540. doi: 10.3389/fpls.2025.1585540 (PMC12267226; doi:10.3389/fpls.2025.1585540)
Supplement: Supplementary file 1 [file DataSheet1.pdf]

Supplementary Material

Overview of tables and figures

Table S1. List of *Vanilla* species naturally occurring in the Neotropical realm, the amount of georeferenced occurrence records found for each species (after spatial filtering), and the known pollination mechanisms and pollinator species (in case of animal-mediated pollination). *Vanilla* species highlighted in grey had enough occurrence records (≥ 30) to perform accurate species distribution modelling.

Table S2. Sources for georeferenced *Vanilla* and pollinator species occurrence records.

Table S3. Climate, soil, and topography variables used as predictors in the MaxEnt

Table S4. Area under the receiving operating characteristic (ROC) curve (AUC) values of the MaxEnt models for the 11 *Vanilla* (ones with enough occurrence records to perform accurate species distribution modelling) and 7 *Vanilla* pollinator species, and the two most important predictor variables explaining the distribution of the corresponding species.

Table S5. Permutation importance (%) of the climate, soil and topography variables used as predictors in the MaxEnt modelling of the 11 *Vanilla* species.

Table S6. Permutation importance (%) of the climate variables used as predictors in the MaxEnt modelling of the 7 pollinator species.

Table S7. Predicted changes (%) in area of suitable habitat by 2050 under the "middle of the road" (SSP2-4.5) and "regional rivalry" (SSP3-7.0) scenarios for the 11 modelled *Vanilla* species and 7 pollinator species. Calculations were made considering the area encompassed by the convex hulls around the presence points of the species (see 2.1.3).

Fig. S1. Presence-absence maps of the modelled *Vanilla* species under current climate conditions (left), and for the year 2050 under SSP2-4.5 (middle) and SSP3-7.0 (right) scenarios. Dark brown indicates suitable areas under both present and future climate conditions. Blue and light brown indicate, respectively, loss (i.e. contraction) and gain in suitable habitat (i.e. expansion) by 2050.

Fig. S2. Presence-absence maps of the modelled pollinator species under current climate conditions (left), and for the year 2050 under SSP2-4.5 (middle) and SSP3-7.0 (right) scenarios. Dark brown indicates suitable areas under both present and future climate conditions. Blue and light brown indicate, respectively, loss (i.e. contraction) and gain in suitable habitat (i.e. expansion) by 2050.

Table S1. List of *Vanilla* species naturally occurring in the Neotropical realm, the amount of georeferenced occurrence records found for each species (after spatial filtering), and the known pollination mechanisms and pollinator species (in case of animal-mediated pollination). Vanilla species highlighted in grey had enough occurrence records (≥ 30) to perform accurate species distribution modelling.

	**Species**	**Georeferenced occurrence records**	**Pollination mechanism**	**Pollinator group**	**Reference**
Vanilla subg. *Vanilla*	*Vanilla acuta* Rolfe	0	unknown		
	Vanilla angustipetala Schltr.	6	unknown		
	Vanilla arcuate Pansarin & M.R. Miranda	2	unknown		
	Vanilla armoriquensis Damián & Mitidieri	0	unknown		
	Vanilla bertoniensis Bertoni	0	unknown		
	Vanilla bradei Schltr. ex Mansf.	0	unknown		
	Vanilla costaricensis Soto Arenas	5	unknown		
	Vanilla dietschiana Edwall	1	unknown		
	Vanilla edwallii Hoehne	21	animal-pollinated	Centridini	Pansarin et al. 2013
	Vanilla guianensis Splitg.	22	autogamous	n.a.	Soto Arenas 2003; Householder et al. 2010
	***Vanilla inodora* Schiede**	**40**	**autogamous**	**n.a.**	**Soto Arenas & Dressler 2010**
	Vanilla methonica Rchb.f. & Warsz.	4	unknown		
	Vanilla mexicana Mill.	**59**	**autogamous**	**n.a.**	**Gigant et al. 2016**
	Vanilla organensis Rolfe	11	unknown		
	Vanilla oroana Dodson	3	unknown		
	Vanilla parvifolia Soto Arenas	2	unknown		
	Vanilla sarapiquensis Soto Arenas	3	unknown		
	Vanilla verrucosa Hauman	2	unknown		
Vanilla sect. *Tethya*	*Vanilla bakeri* Schltr.	0	unknown		
	Vanilla barbellata Rchb.f.	23	animal-pollinated	Centridini	Nielsen & Ackerman, unpubl.
	Vanilla claviculata Sw.	7	animal-pollinated	Centridini	Nielsen & Ackerman, unpubl.
	Vanilla dilloniana Correll	29	animal-pollinated	Centridini	Nielsen & Ackerman, unpubl.
	Vanilla marmoreisensis Soto Calvo, Esperon & Sauleda	0	unknown		
	Vanilla poitaei Rchb.f.	22	unknown		
Vanilla sect. *Xanata*	*Vanilla appendiculata* Rolfe	21	unknown		
	Vanilla bicolor Lindl.	**40**	**autogamous**	**n.a.**	**Householder et al. 2010; van Dam et al. 2010**
	Vanilla capixaba Fraga & D.R.Couto	1	unknown		
	Vanilla carinata Rolfe	0	unknown		
	Vanilla chamissonis Klotzsch	**62**	**autogamous**	**n.a.**	**Reis 2000; Rodolphe et al. 2011**
	Vanilla cobanensis Archila	0	unknown		
	Vanilla columbiana Rolfe	17	unknown		
	Vanilla corinnae Sambin & Chiron	1	unknown		
	Vanilla cribbiana Soto Arenas	12	animal-pollinated	Euglossini	Soto Arenas & Dressler 2010
	Vanilla dressleri Soto Arenas	26	animal-pollinated	Euglossini	Soto Arenas & Dressler 2010
	Vanilla dubia Huber	6	animal-pollinated	Euglossini	Pansarin & Pansarin 2014
	Vanilla duckei Hoehne	1	unknown		

Vanilla dungsii Pabst	0	unknown		
Vanilla espondae Soto Arenas	1	unknown		
Vanilla fimbriata Rolfe	0	unknown		
***Vanilla hartii* Rolfe**	**47**	**animal-pollinated**	**Euglossini**	**Watteyn et al. 2023**
Vanilla helleri A.D.Hawkes	2	unknown		
Vanilla hostmannii Rolfe	29	unknown		
Vanilla inornata Sambin & Chiron	2	unknown		
Vanilla insignis Ames	24	animal-pollinated	Euglossini	Soto Arenas & Dressler 2010
Vanilla javieri Bar.-Colm.	0	unknown		
Vanilla karen-christianae Karremans & P.Lehm.	4	unknown		
Vanilla labellopapillata A.K.Koch, Fraga J.U.Santos & Ilk.-Borg.	3	unknown		
Vanilla marowynensis Pulle	3	unknown		
***Vanilla odorata* C.Presl**	**98**	**animal-pollinated**	**Euglossini**	**Personal observations**
***Vanilla palmarum* Lindl**	**226**	**autogamous**	**n.a.**	**Householder et al. 2010; Soto Arenas & Cribb 2013**
Vanilla paulista Fraga & Pansarin	0	unknown		
Vanilla penicillata Garay & Dunst.	5	unknown		
***Vanilla phaeantha* Rchb.f.**	**204**	**animal-pollinated**	**Euglossini**	**Anjos et al. 2017**
***Vanilla planifolia* Andrews**	**143**	**animal-pollinated**	**Euglossini Meliponini**	**Soto Arenas & Dressler 2010; Pemberton et al. 2023 Karremans 2024**
***Vanilla pompona* Schiede**	**171**	**animal-pollinated**	**Euglossini**	**Ackerman 1983; Lubinsky et al. 2006; Householder et al. 2010; Watteyn et al. 2022**
Vanilla ribeiroi Hoehne	5	unknown		
Vanilla rivasii Molineros, R.T.González, Flanagan & J.T.Otero	1	unknown		
Vanilla ruiziana Klotzsch	5	unknown		
Vanilla sprucei Rolfe	19	unknown		
Vanilla tahitensis J.W.Moore	0	unknown		
***Vanilla trigonocarpa* Hoehne**	**68**	**animal-pollinated**	**Euglossini**	**Soto Arenas & Dressler 2010; Personal observations**
Vanilla vellozoi Rolfe	0	unknown		
Vanilla weberbaueriana J.W.Moore	1	unknown		

Table S2. Sources for georeferenced *Vanilla* and pollinator species occurrence records.

Taxa	Source	Reference
Vanilla spp.	Global Biodiversity Information Facility (GBIF)	GBIF (2024). Available from: https://www.gbif.org<br>Chamberlain S, Barve V, Mcglinn D, Oldoni D, Desmet P, Geffert L, Ram K (2024). rgbif: Interface to the Global Biodiversity Information Facility API. R package version 3.8.1, https://CRAN.R-project.org/package=rgbif
	Botanical Information and Ecology Network (BIEN)	BIEN (2024). Available from : https://bien.nceas.ucsb.edu/bien/biendata<br>Maitner, B.S., Boyle, B., Casler, N., Condit, R., Donoghue, J., Durán, S.M., Guaderrama, D., Hinchliff, C.E., Jørgensen, P.M., Kraft, N.J. and McGill, B., 2018. The bien r package: A tool to access the Botanical Information and Ecology Network (BIEN) database. Methods in Ecology and Evolution, 9(2), pp.373-379
	Herbarium specimen	Georeferenced data available from:<br>Herbario AMO (https://www.herbarioamo.org)<br>Missouri Botanic Garden (https://www.tropicos.org)<br>Lankester Botanic Garden
	Literature	Georeferenced data available in:<br>Soto Arenas, M.A. and Dressler, R.L., 2010. A revision of the Mexican and Central American species of Vanilla Plumier ex Miller with a characterization of their ITS region of the nuclear ribosomal DNA. *Lankesteriana*, *9*(3), pp.285-354<br>Karremans, A.P., Chinchilla, I.F., Rojas-Alvarado, G., Cedeño-Fonseca, M., Damián, A. and Léotard, G., 2020. A reappraisal of neotropical Vanilla. With a note on taxonomic inflation and the importance of alpha taxonomy in biological studies. *Lankesteriana*, *20*(3), pp.395-497
Bee spp.	Global Biodiversity Information Facility (GBIF)	GBIF (2024). Available from: https://www.gbif.org.<br>Chamberlain S, Barve V, Mcglinn D, Oldoni D, Desmet P, Geffert L, Ram K (2024). rgbif: Interface to the Global Biodiversity Information Facility API. R package version 3.8.1, https://CRAN.R-project.org/package=rgbif.
	Literature	Georeferenced data available in:<br>Ramírez, S.R., Hernández, C., Link, A. and López-Uribe, M.M., 2015. Seasonal cycles, phylogenetic assembly, and functional diversity of orchid bee communities. *Ecology and evolution*, *5*(9), pp.1896-1907<br>Faleiro, F.V., Nemésio, A. and Loyola, R., 2018. Climate change likely to reduce orchid bee abundance even in climatic suitable sites. *Global Change Biology*, *24*(6), pp.2272-2283<br>Opedal, Ø.H., Martins, A.A. and Marjakangas, E.L., 2020. A database and synthesis of euglossine bee assemblages collected at fragrance baits. *Apidologie*, *51*(4), pp.519-530<br>Dorey, J.B., Fischer, E.E., Chesshire, P.R., Nava-Bolaños, A., O'Reilly, R.L., Bossert, S., Collins, S.M., Lichtenberg, E.M., Tucker, E.M., Smith-Pardo, A. and Falcon-Brindis, A., 2023. A globally synthesised and flagged bee occurrence dataset and cleaning workflow. *Scientific Data*, *10*(1), p.747

Table S3. Climate, soil, and topography variables used as predictors in the MaxEnt models.

Variable	Code	Reference
Annual mean temperature	bio1	Fick, S.E. and Hijmans, R.J., 2017. WorldClim 2: new 1-km spatial resolution climate surfaces for global land areas. *International journal of climatology*, *37*(12), pp.4302-4315
Mean diurnal range	bio2	
Isothermality	bio3	
Temperature seasonality	bio4	
Maximum temperature of warmest month	bio5	
Minimum temperature of coldest month	bio6	
Temperature annual range	bio7	
Mean Temperature of Warmest Quarter	bio10	
Mean Temperature of Coldest Quarter	bio11	
Annual precipitation	bio12	
Precipitation of Wettest Month	bio13	
Precipitation of Driest Month	bio14	
Precipitation seasonality	bio15	
Precipitation of wettest quarter	bio16	
Precipitation of driest quarter	bio17	
Available soil water capacity	AWCh2	Hengl, T., Mendes de Jesus, J., Heuvelink, G.B., Ruiperez Gonzalez, M., Kilibarda, M., Blagotić, A., Shangguan, W., Wright, M.N., Geng, X., Bauer-Marschallinger, B. and Guevara, M.A., 2017. SoilGrids250m: Global gridded soil information based on machine learning. *PLoS one*, *12*(2), p.e0169748
Bulk density	BLDFIE	
Cation exchange capacity of soil	CECSOL	
Weight percentage of the clay particles	CLYPPT	
Soil organic carbon content	ORCDRC	
pH index measured in water solution	PHIHOX	
Weight percentage of the slit particles	SLTPPT	
weight percentage of the sand particles	SNDPPT	
Slope	Slope	Abrams, M., Yamaguchi, Y. and Crippen, R., 2022. Aster global dem (gdem) version 3. *The International Archives of the Photogrammetry, Remote Sensing and Spatial Information Sciences*, *43*, pp.593-598.
Topographic position index	TPI	
Terrain ruggedness index	TRI	
Topographic wetness index	TWO	

Table S4. Area under the receiving operating characteristic (ROC) curve (AUC) values of the MaxEnt models for the 11 *Vanilla* (ones with enough occurrence records to perform accurate species distribution modelling) and 7 *Vanilla* pollinator species, and the two most important predictor variables explaining the distribution of the corresponding species.

	**Species**	**AUC ± SD***	**Variable1**	**Variable2**
Vanilla	*Vanilla bicolor*	0.82 ± 0.04	Bio14	PHIHOX
	Vanilla chamissonis	0.83 ± 0.12	bio7	bio11
	Vanilla hartii	0.92 ± 0.04	bio12	bio15
	Vanilla inodora	0.86 ± 0.08	bio7	bio6
	Vanilla mexicana	0.76 ± 0.09	bio14	bio11
	Vanilla odorata	0.83 ± 0.04	bio6	bio15
	Vanilla palmarum	0.85 ± 0.04	bio6	bio4
	Vanilla phaeantha	0.87 ± 0.07	bio12	PHIHOX
	Vanilla planifolia	0.83 ± 0.07	bio4	bio7
	Vanilla pompona	0.82 ± 0.06	bio6	PHIHOX
	Vanilla trigonocarpa	0.81 ± 0.08	bio15	bio13
Pollinators	*Euglossa asarophora*	0.76 ± 0.13	bio11	bio7
	Euglossa cybelia	0.77 ± 0.15	bio2	bio13
	Euglossa dilemma	0.81 ± 0.18	bio11	bio6
	Euglossa tridentata	0.84 ± 0.05	bio1	bio4
	Eulaema cingulata	0.81 ± 0.05	bio6	bio12
	Eulaema meriana	0.89 ± 0.03	bio1	bio4
	Eulaema nigrita	0.86 ± 0.04	bio11	bio15

*standard deviation

Table S5. Permutation importance (%) of the climate, soil and topography variables used as predictors in the MaxEnt modelling of the 11 *Vanilla* species.

**Variable**	***V. bicolor***	***V. chamissonis***	***V. hartii***	***V. inodora***	***V. mexicana***	***V. odorata***	***V. palmarum***	***V. phaeantha***	***V. planifolia***	***V. pompona***	***V. trigonocarpa***
bio1	0.00	0.00	0.00	4.00	0.00	2.62	0.45	1.37	0.40	4.63	0.00
bio2	0.00	12.03	0.00	10.32	11.13	1.94	1.49	9.31	0.28	0.63	0.00
bio3	2.76	13.52	5.86	0.00	0.00	0.00	14.80	5.60	1.21	3.46	4.04
bio4	13.01	0.00	6.96	7.67	1.64	7.27	16.96	0.44	25.79	2.54	0.00
bio5	0.00	0.00	0.78	0.00	0.00	0.00	0.87	0.00	0.00	0.00	0.00
bio6	0.00	0.37	0.00	13.70	0.00	30.98	22.57	0.00	3.67	29.83	0.00
bio7	0.96	22.54	0.00	33.77	0.00	0.00	5.69	1.42	12.95	7.01	0.00
bio10	0.22	0.00	0.00	5.81	0.00	0.00	0.13	0.00	0.89	0.01	4.91
bio11	0.14	21.18	0.00	0.00	24.24	0.00	0.00	14.97	0.00	0.00	5.78
Temperature	17.08	69.64	13.60	75.27	37.01	42.80	62.96	33.12	45.19	48.12	14.73
bio12	7.40	0.00	21.46	0.28	1.54	0.00	3.24	23.06	10.58	0.00	0.00
bio13	0.00	0.00	14.64	9.08	0.00	6.04	0.84	9.05	6.80	6.90	15.96
bio14	18.21	0.00	0.00	0.08	44.80	2.74	0.17	0.27	2.29	1.57	0.00
bio15	7.28	0.00	19.17	0.00	0.00	15.96	6.45	0.00	0.98	8.87	35.14
bio16	0.00	8.45	0.00	0.81	0.00	0.00	0.00	0.00	4.95	0.33	0.43
bio17	0.00	0.00	12.80	0.01	0.00	8.16	14.91	0.00	1.70	1.60	5.08
Precipitation	32.89	8.45	68.07	10.26	44.80	32.90	25.62	32.39	27.31	19.26	56.61
**Climate**	**49.98**	**78.09**	**81.67**	**85.53**	**81.81**	**75.70**	**88.58**	**65.51**	**72.50**	**67.38**	**71.34**
AWCh2	5.16	0.00	0.04	0.65	0.00	0.00	0.82	0.07	0.10	0.01	0.00
BLDFIE	3.71	16.35	0.00	0.12	0.00	0.78	0.00	0.00	0.20	0.51	0.11
CECSOL	6.91	0.42	7.90	0.58	0.0	0.15	2.09	0.00	1.07	0.18	7.87
CLYPPT	4.39	0.00	6.67	0.00	0.00	0.00	2.98	0.02	4.68	0.95	0.00
ORCDRC	4.14	2.83	0.00	0.27	14.12	0.00	0.00	0.94	2.76	0.97	0.00
PHIHOX	17.57	0.00	0.10	7.60	0.00	6.11	1.69	21.43	9.27	9.73	8.29
SLTPPT	0.79	0.00	1.78	0.00	2.53	3.92	0.40	0.15	1.55	5.08	0.00
SNDPPT	0.00	2.31	0.00	1.54	0.00	11.59	0.00	10.66	4.60	1.22	0.04
**Soil**	**42.66**	**21.91**	**16.47**	**10.76**	**16.65**	**22.55**	**7.98**	**33.27**	**24.24**	**18.64**	**16.30**
TWI	1.68	0.00	0.00	2.89	0.00	1.14	0.00	1.03	0.34	7.89	12.11
slope	0.16	0.00	0.00	0.00	0.00	0.25	0.00	0.00	2.93	4.37	0.00
TPI	0.00	0.00	1.86	0.17	0.00	0.15	0.00	0.20	0.00	0.00	0.21
TRI	5.55	0.00	0.00	0.65	0.00	0.22	3.44	0.00	0.00	1.72	0.04
**Topography**	**7.38**	**0.00**	**1.86**	**3.71**	**0.00**	**1.75**	**3.44**	**1.23**	**3.27**	**13.98**	**12.35**

Table S6. Permutation importance (%) of the climate variables used as predictors in the MaxEnt modelling of the 7 pollinator species.

**Variable**	***Euglossa asarophora***	***Euglossa cybelia***	***Euglossa dilemma***	***Euglossa tridentata***	***Eulaema cingulata***	***Eulaema meriana***	***Eulaema nigrita***
bio1	0.00	0.00	1.37	34.94	0.10	30.59	1.37
bio2	23.58	36.32	3.51	0.00	0.58	0.00	2.17
bio3	8.22	0.00	4.47	2.21	0.00	1.98	1.45
bio4	1.56	0.00	7.28	15.74	4.52	23.89	8.49
bio5	0.00	0.00	0.00	0.00	1.44	3.84	7.47
bio6	0.00	0.00	12.24	9.77	53.81	5.36	5.26
bio7	12.05	14.92	1.20	14.61	0.00	0.00	0.04
bio10	11.29	0.00	2.17	3.55	2.23	1.32	7.04
bio11	30.95	9.33	30.13	6.11	0.00	0.46	38.72
**Temperature**	**87.64**	**51.24**	**62.37**	**86.92**	**62.68**	**67.44**	**72.01**
bio12	0.00	0.00	16.62	0.00	29.29	2.48	2.71
bio13	0.90	25.77	10.99	2.16	0.00	0.07	0.69
bio14	0.68	0.00	3.69	0.00	0.14	0.30	4.43
bio15	0.00	11.18	3.84	5.92	4.07	6.05	12.44
bio16	0.00	2.48	2.02	3.10	3.81	23.67	7.42
bio17	10.78	0.00	0.48	1.91	0.00	0.00	0.31
**Precipitation**	**12.36**	**39.43**	**37.63**	**13.09**	**37.32**	**32.56**	**27.99**

Table S7. Predicted changes (%) in area of suitable habitat by 2050 under the "middle of the road" (SSP2-4.5) and "regional rivalry" (SSP3-7.0) scenarios for the 11 modelled *Vanilla* species and 7 pollinator species. Calculations were made considering the area encompassed by the convex hulls around the presence points of the species (see 2.1.3).

	**Species**	**Present suitable area (km^2)**	**Scenario**	**Range expansion (%)**	**Range contraction (%)**	**Net change (%)**
Vanilla	*Vanilla bicolor*	556,889	SSP2-4.5	31.0	9.3	+ 21.8
			SSP3-7.0	40.0	7.8	+ 32.2
	Vanilla chamissonis	1,309,042	SSP2-4.5	38.4	18.0	+ 20.4
			SSP3-7.0	48.6	18.8	+ 29.8
	Vanilla hartii	604,198	SSP2-4.5	2.3	26.2	- 23.9
			SSP3-7.0	1.1	35.3	- 37.1
	Vanilla inodora	244,675	SSP2-4.5	8.3	8.9	- 0.6
			SSP3-7.0	8.8	11.7	- 2.9
	Vanilla mexicana	5,432,826	SSP2-4.5	12.8	0.5	+ 12.3
			SSP3-7.0	11.8	0.5	+ 11.3
	Vanilla odorata	4,325,207	SSP2-4.5	31.2	0.0	+ 31.2
			SSP3-7.0	35.9	0.1	+ 35.9
	Vanilla palmarum	2,768,244	SSP2-4.5	17.4	43.4	- 26.0
			SSP3-7.0	19.5	47.8	- 28.3
	Vanilla phaeantha	1,757,585	SSP2-4.5	31.1	4.3	+ 26.8
			SSP3-7.0	29.0	7.1	+ 21.9
	Vanilla planifolia	1,112,188	SSP2-4.5	20.8	1.1	+ 19.6
			SSP3-7.0	21.6	2.2	+ 19.4
	Vanilla pompona	4,698,574	SSP2-4.5	6.6	52.5	- 45.9
			SSP3-7.0	6.7	59.5	- 52.8
	Vanilla trigonocarpa	1,251,335	SSP2-4.5	139.9	0.0	+ 139.9
			SSP3-7.0	139.1	0.1	+ 139.0
Vanilla pollinators	*Euglossa asarophora*	174,982	SSP2-4.5	0.7	67.5	- 66.8
			SSP3-7.0	0.7	68.6	- 67.9
	Euglossa cybelia	753,031	SSP2-4.5	9.2	33.8	- 24.6
			SSP3-7.0	8.1	39.8	- 31.7
	Euglossa dilemma	241,119	SSP2-4.5	5.9	74.1	- 68.2
			SSP3-7.0	3.6	74.3	- 70.7
	Euglossa tridentata	2,783,570	SSP2-4.5	0.3	36.0	- 35.7
			SSP3-7.0	0.3	42.4	- 42.2
	Eulaema cingulata	8,085,530	SSP2-4.5	14.8	21.7	- 6.9
			SSP3-7.0	14.2	32.8	- 18.5
	Eulaema meriana	8,193,707	SSP2-4.5	0.4	14.0	- 13.6
			SSP3-7.0	0.5	20.3	- 19.9
	Eulaema nigrita	10,539,410	SSP2-4.5	0.3	27.7	- 27.4
			SSP3-7.0	0.5	32.0	- 31.6

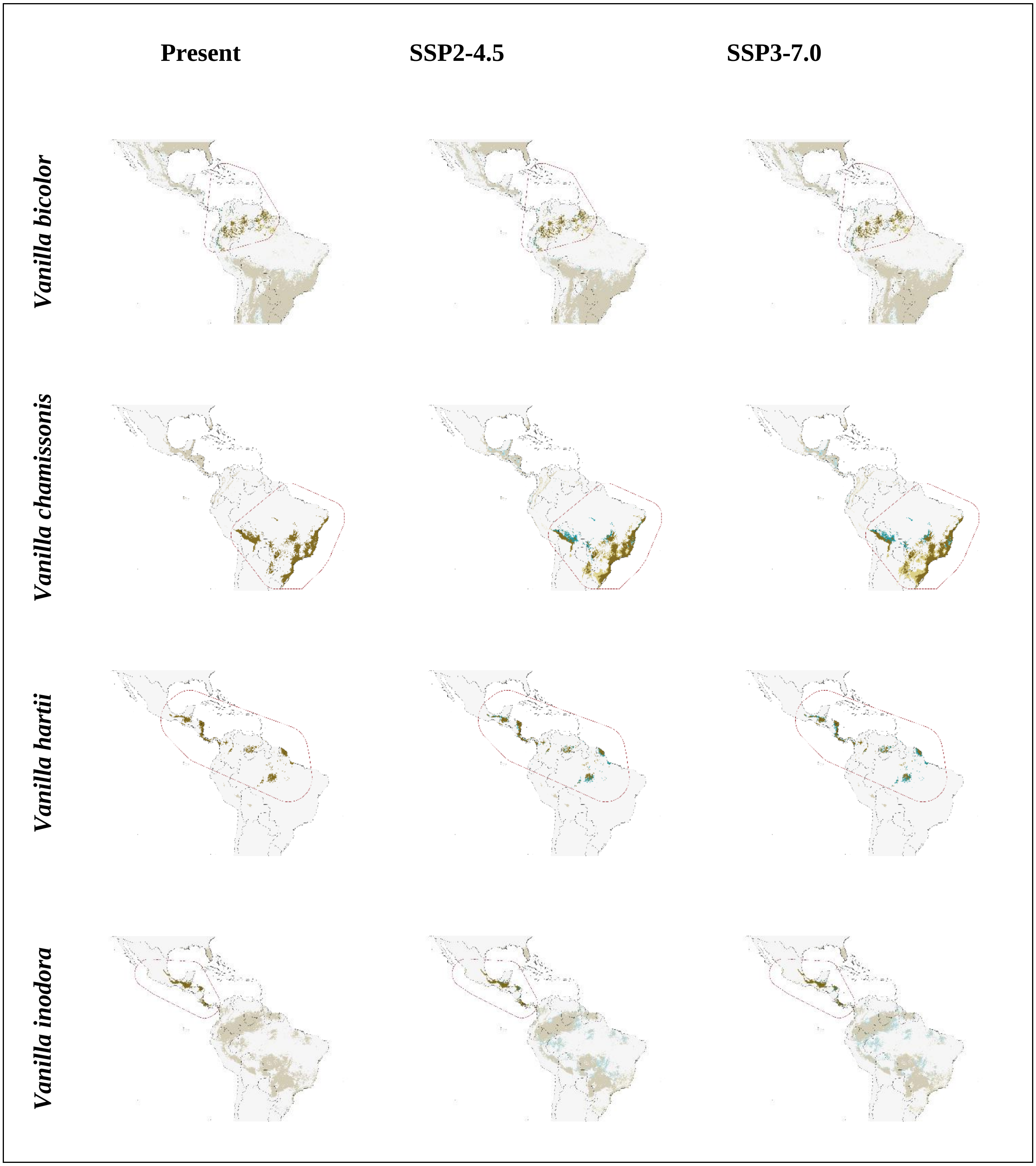
Present
SSP2-4.5
SSP3-7.0
Vanilla bicolor
Vanilla chamissonis
Vanilla hartii
Vanilla inodora

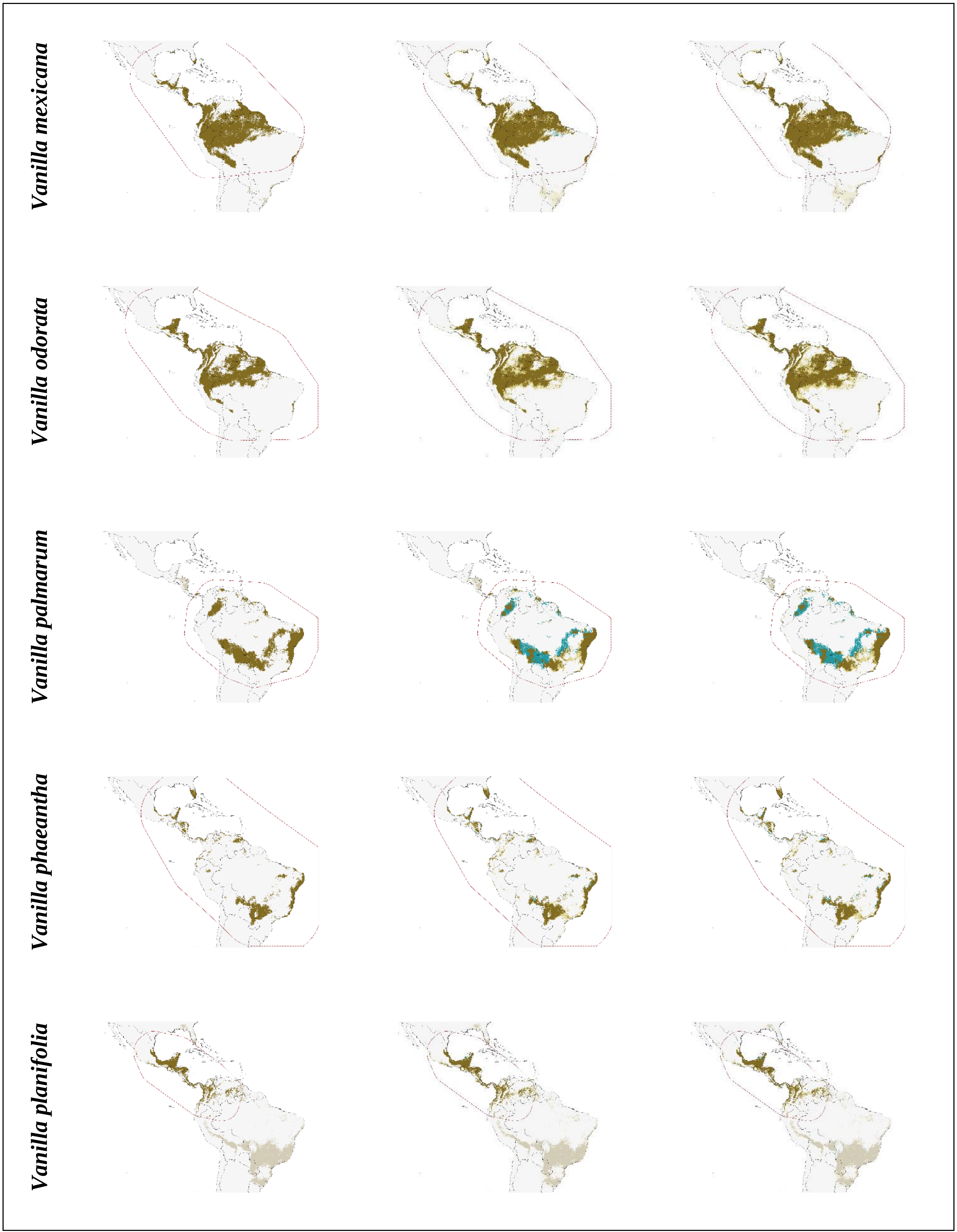
Vanilla mexicana
Vanilla odorata
Vanilla palmarum
Vanilla phaeantha
Vanilla planifolia

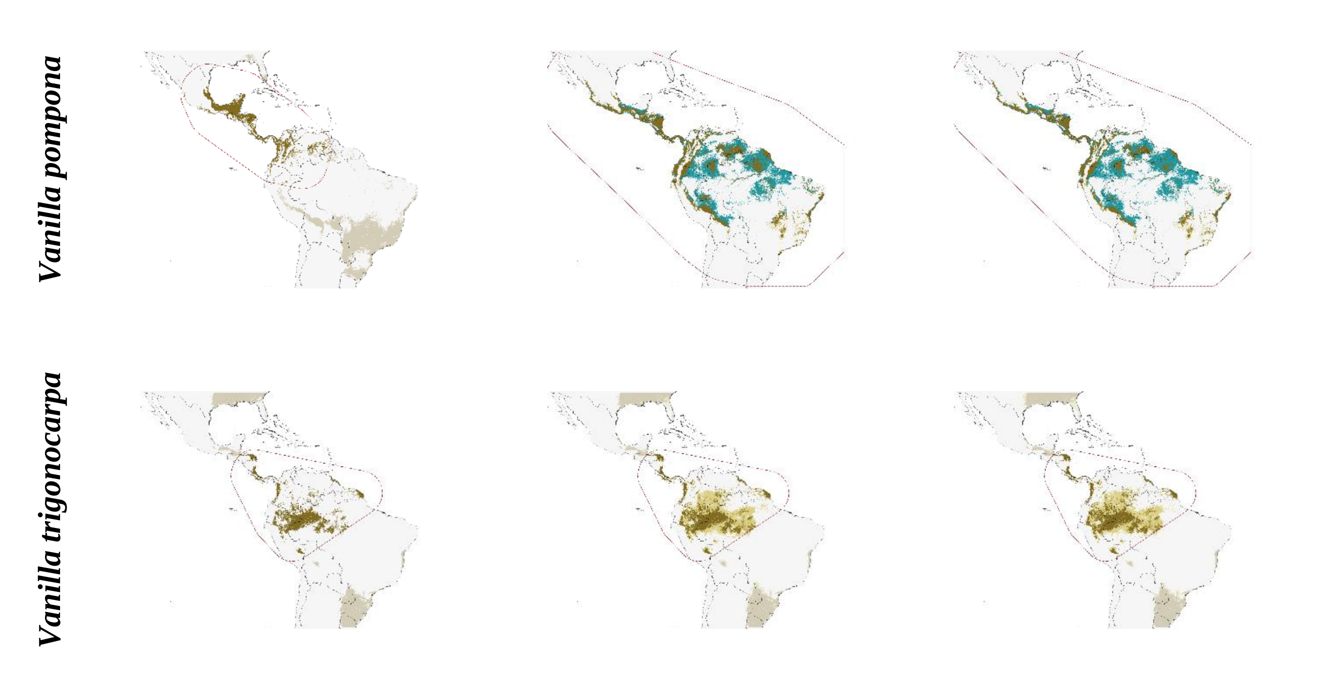

Fig. S1. Presence-absence maps of the modelled *Vanilla* species under current climate conditions (left), and for the year 2050 under SSP2-4.5 (middle) and SSP3-7.0 (right) scenarios. Dark brown indicates suitable areas under both present and future climate conditions. Blue and light brown indicate, respectively, loss (i.e. contraction) and gain in suitable habitat (i.e. expansion) by 2050.

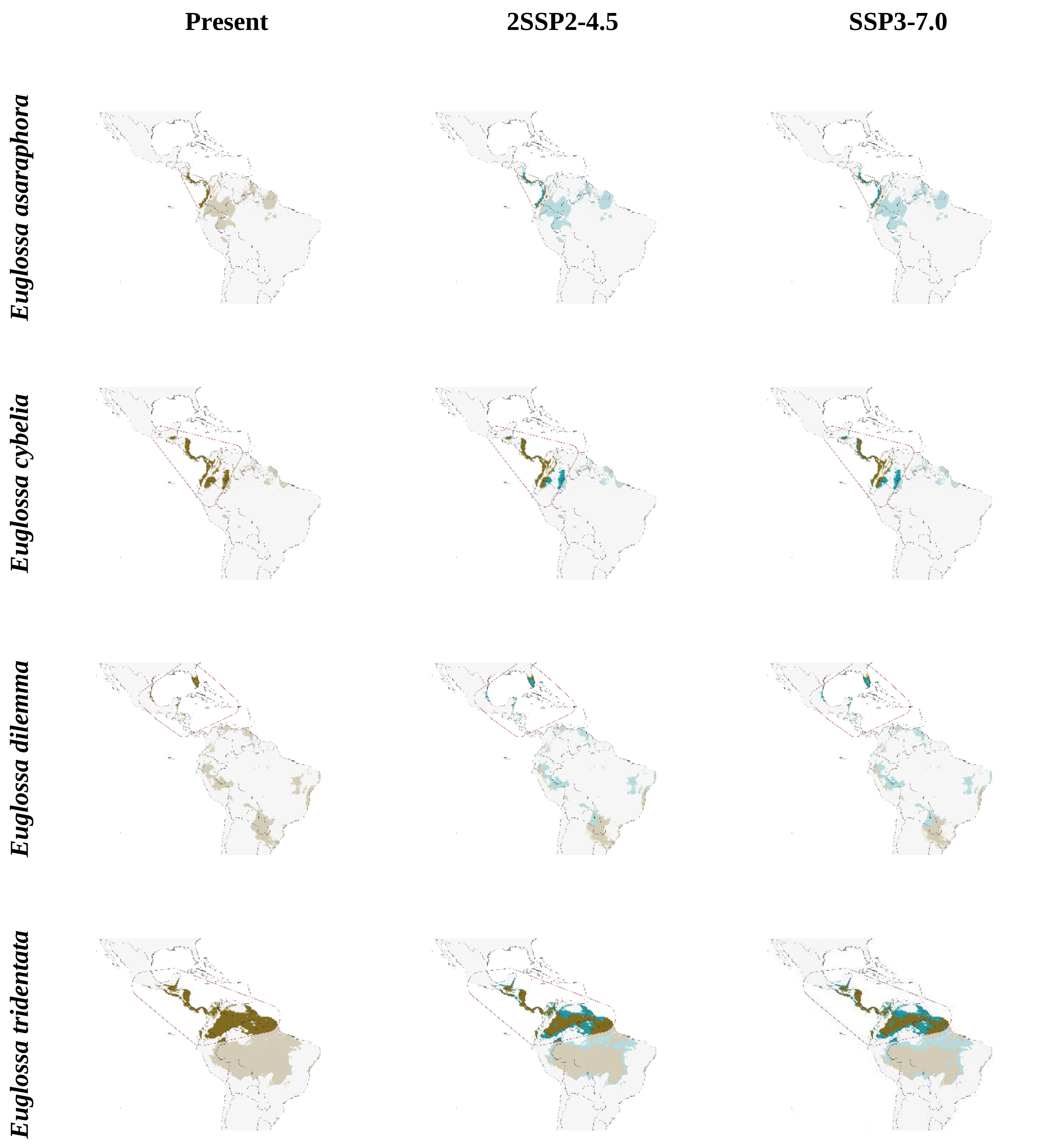
Present
2SSP2-4.5
SSP3-7.0
Euglossa asaraphora
Euglossa cybelia
Euglossa dilemma
Euglossa tridentata

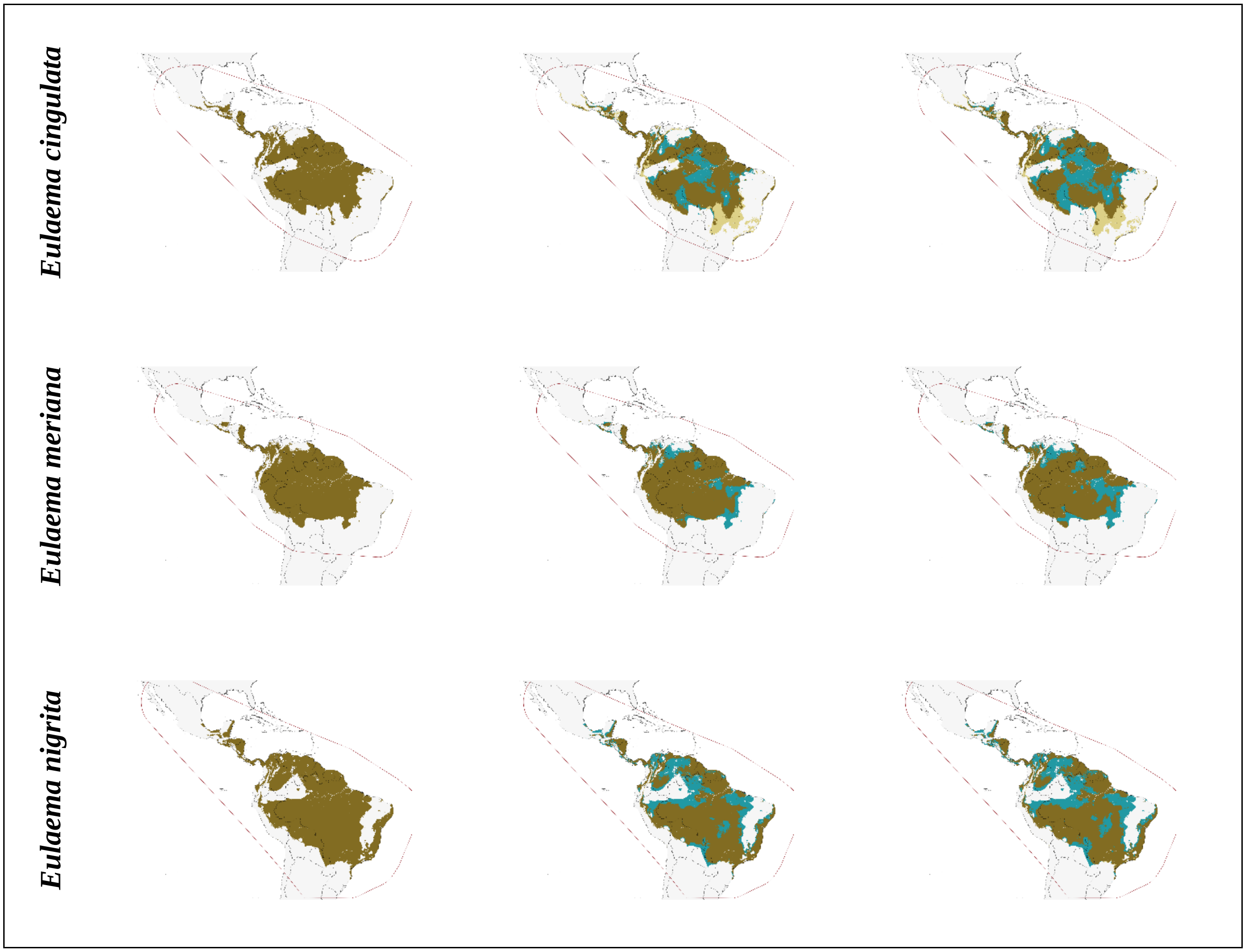

Fig. S2. Presence-absence maps of the modelled pollinator species under current climate conditions (left), and for the year 2050 under SSP2-4.5 (middle) and SSP3-7.0 (right) scenarios. Dark brown indicates suitable areas under both present and future climate conditions. Blue and light brown indicate, respectively, loss (i.e. contraction) and gain in suitable habitat (i.e. expansion) by 2050.
