# Supplementary material for: Wild Vanilla and pollinators at risk of spatial mismatch in a changing climate
Source: Front Plant Sci. 2025 Jul 3;16:1585540. doi: 10.3389/fpls.2025.1585540 (PMC12267226; doi:10.3389/fpls.2025.1585540)
Supplement: Supplementary file 2 [file DataSheet2.pdf]

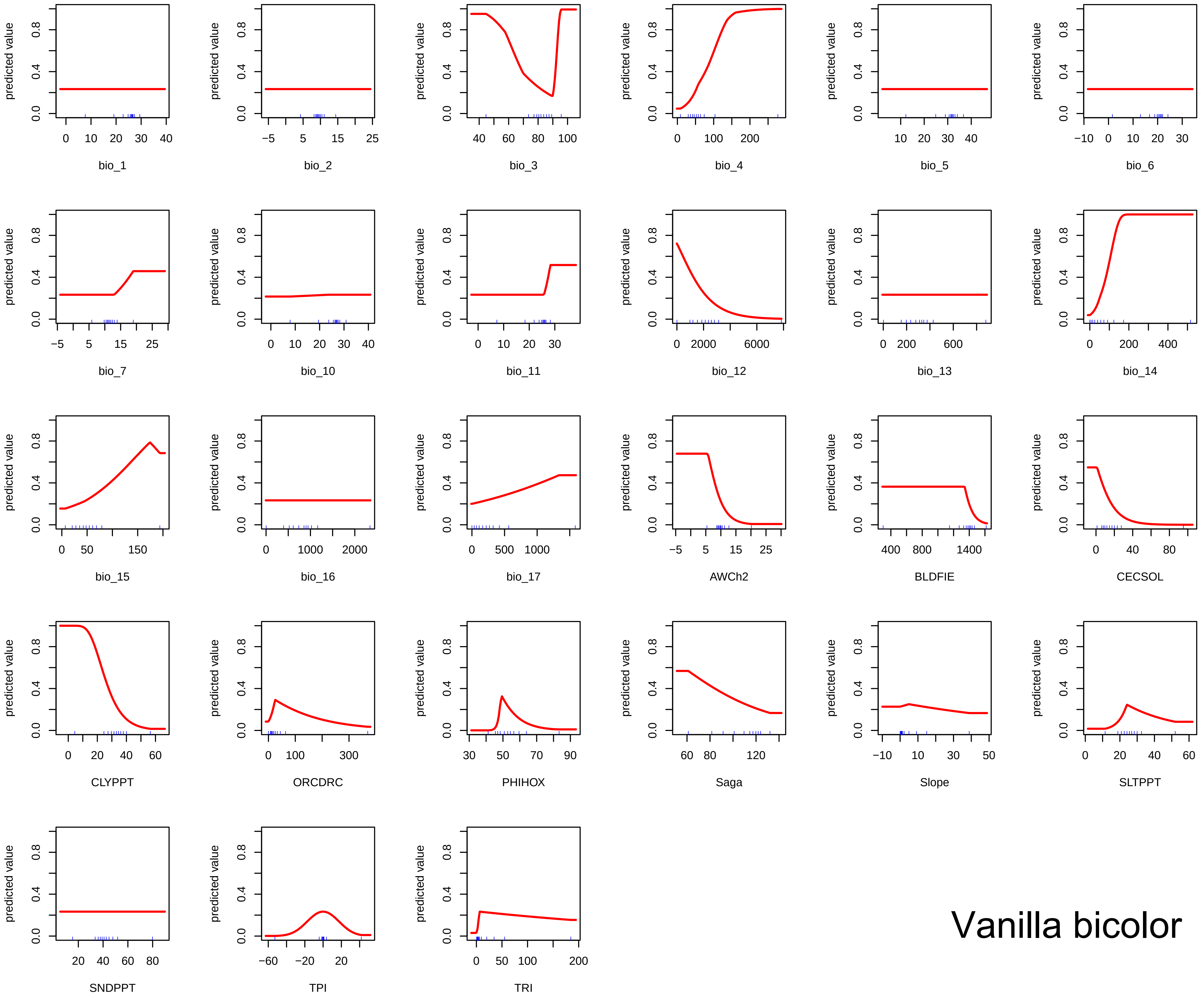
predicted value
bio_1
bio_2
bio_3
bio_4
bio_5
bio_6
bio_7
bio_10
bio_11
bio_12
bio_13
bio_14
bio_15
bio_16
bio_17
AWCh2
BLDFIE
CECSOL
CLYPPT
ORCDRC
PHIHOX
Saga
Slope
SLTPPT
SNDPPT
TPI
TRI
Vanilla bicolor

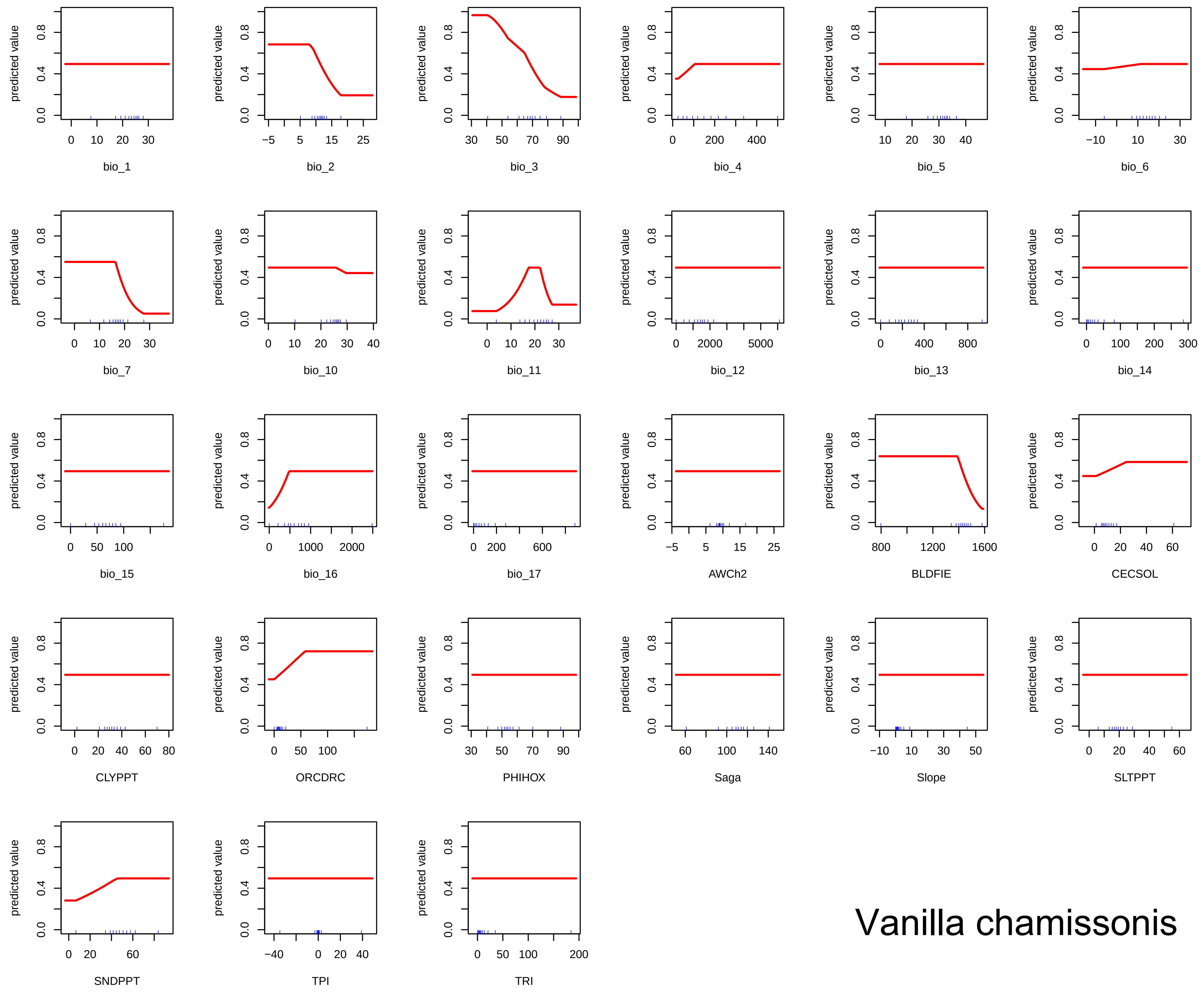

predicted value
bio_1
bio_2
bio_3
bio_4
bio_5
bio_6
bio_7
bio_10
bio_11
bio_12
bio_13
bio_14
bio_15
bio_16
bio_17
AWCh2
BLDFIE
CECSOL
CLYPPT
ORCDRC
PHIHOX
Saga
Slope
SLTPPT
SNDPPT
TPI
TRI
Vanilla chamissonis

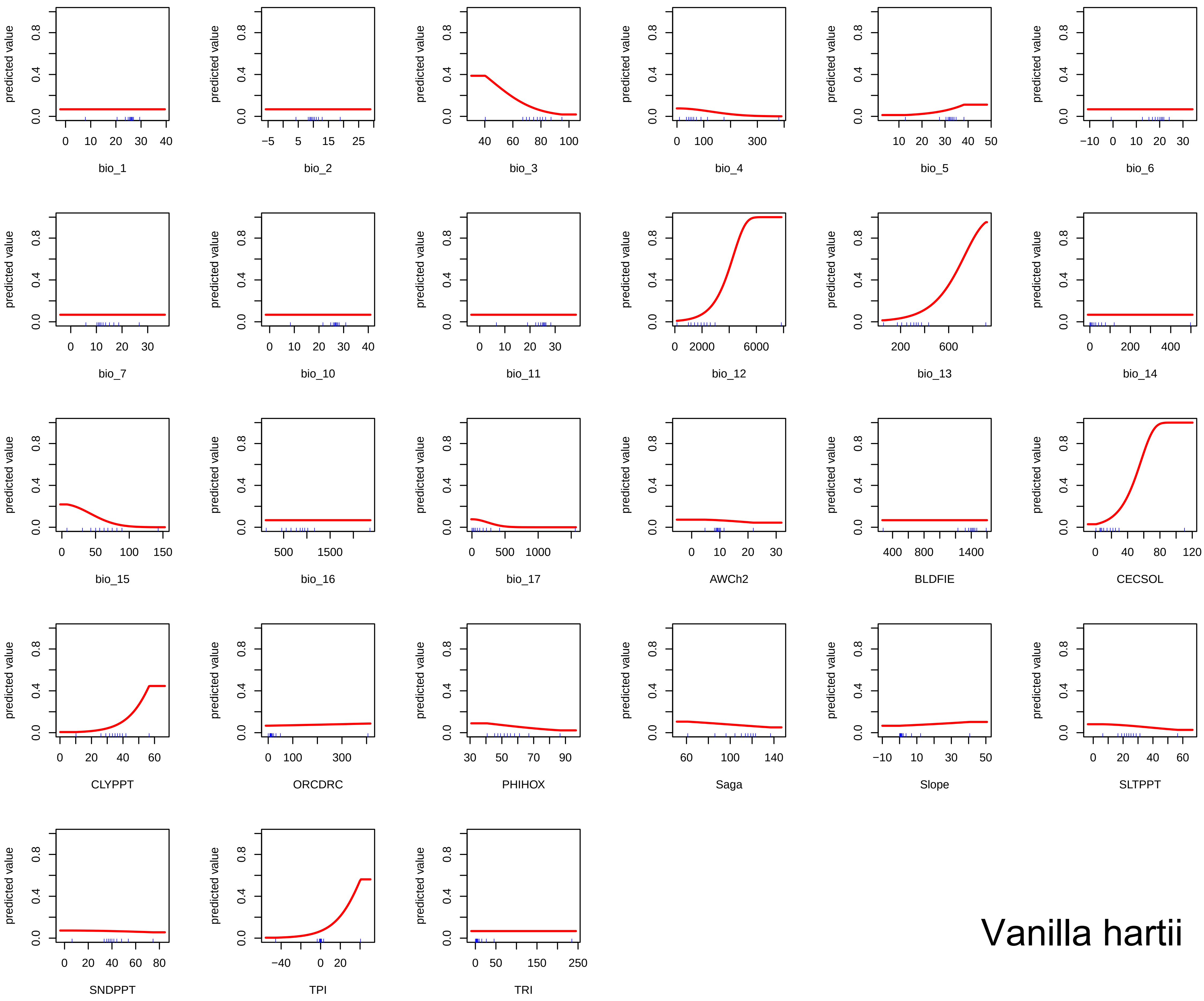
predicted value
bio_1
bio_2
bio_3
bio_4
bio_5
bio_6
bio_7
bio_10
bio_11
bio_12
bio_13
bio_14
bio_15
bio_16
bio_17
AWCh2
BLDFIE
CECSOL
CLYPPT
ORCDRC
PHIHOX
Saga
Slope
SLTPPT
SNDPPT
TPI
TRI
Vanilla hartii

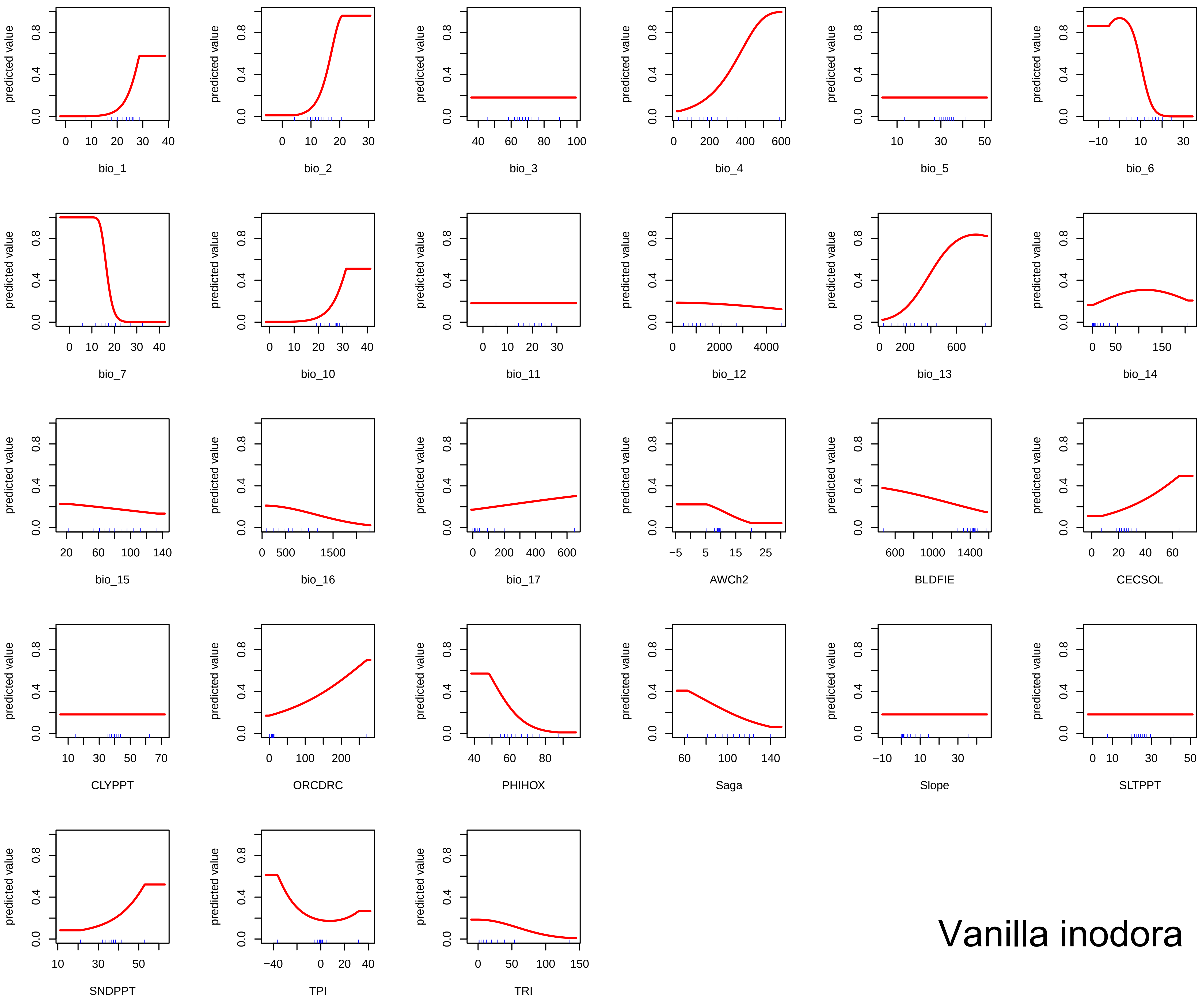
predicted value
bio_1
bio_2
bio_3
bio_4
bio_5
bio_6
bio_7
bio_10
bio_11
bio_12
bio_13
bio_14
bio_15
bio_16
bio_17
AWCh2
BLDFIE
CECSOL
CLYPPT
ORCDRC
PHIHOX
Saga
Slope
SLTPPT
SNDPPT
TPI
TRI
Vanilla inodora

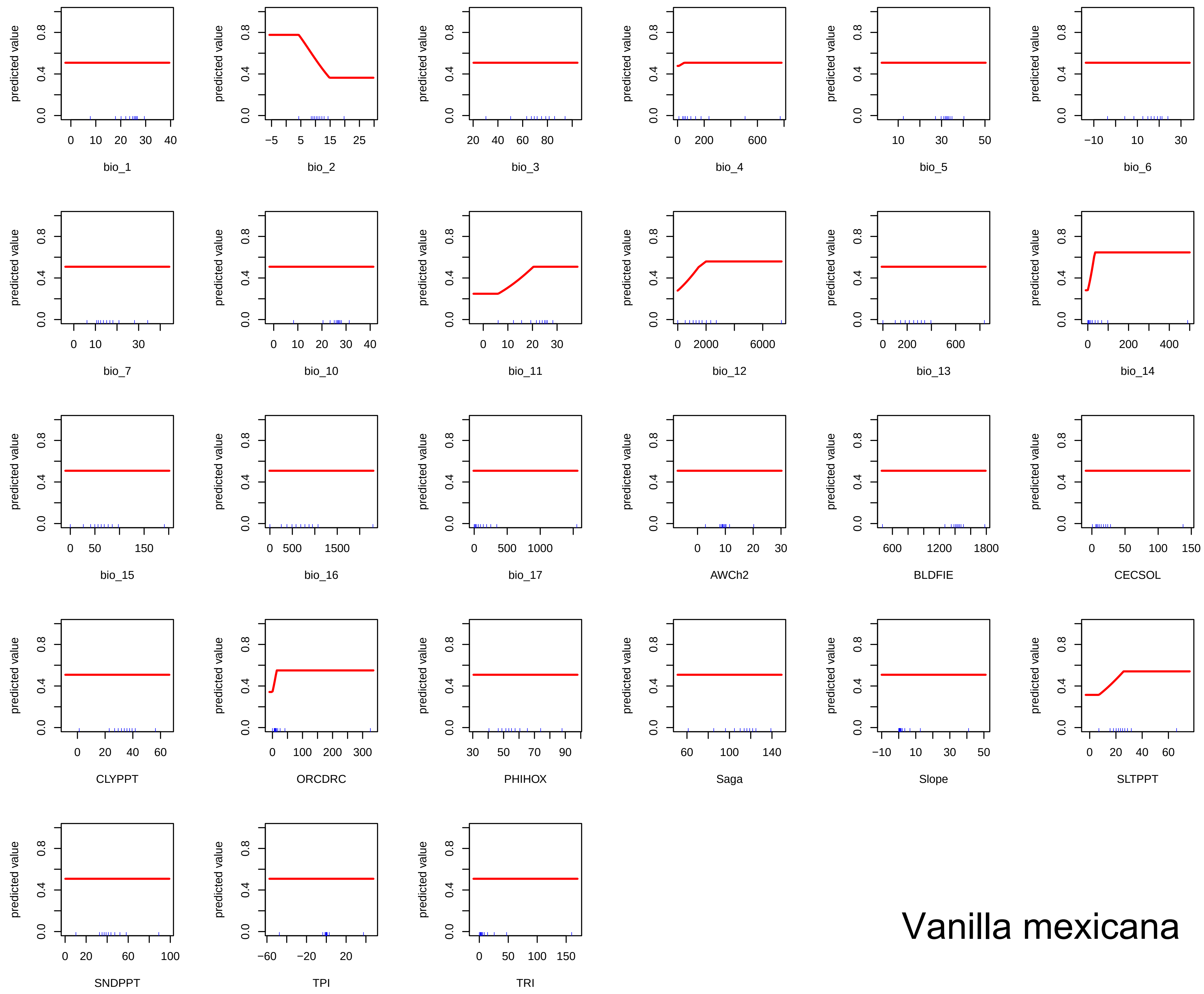
predicted value
bio_1
bio_2
bio_3
bio_4
bio_5
bio_6
bio_7
bio_10
bio_11
bio_12
bio_13
bio_14
bio_15
bio_16
bio_17
AWCh2
BLDFIE
CECSOL
CLYPPT
ORCDRC
PHIHOX
Saga
Slope
SLTPPT
SNDPPT
TPI
TRI
Vanilla mexicana

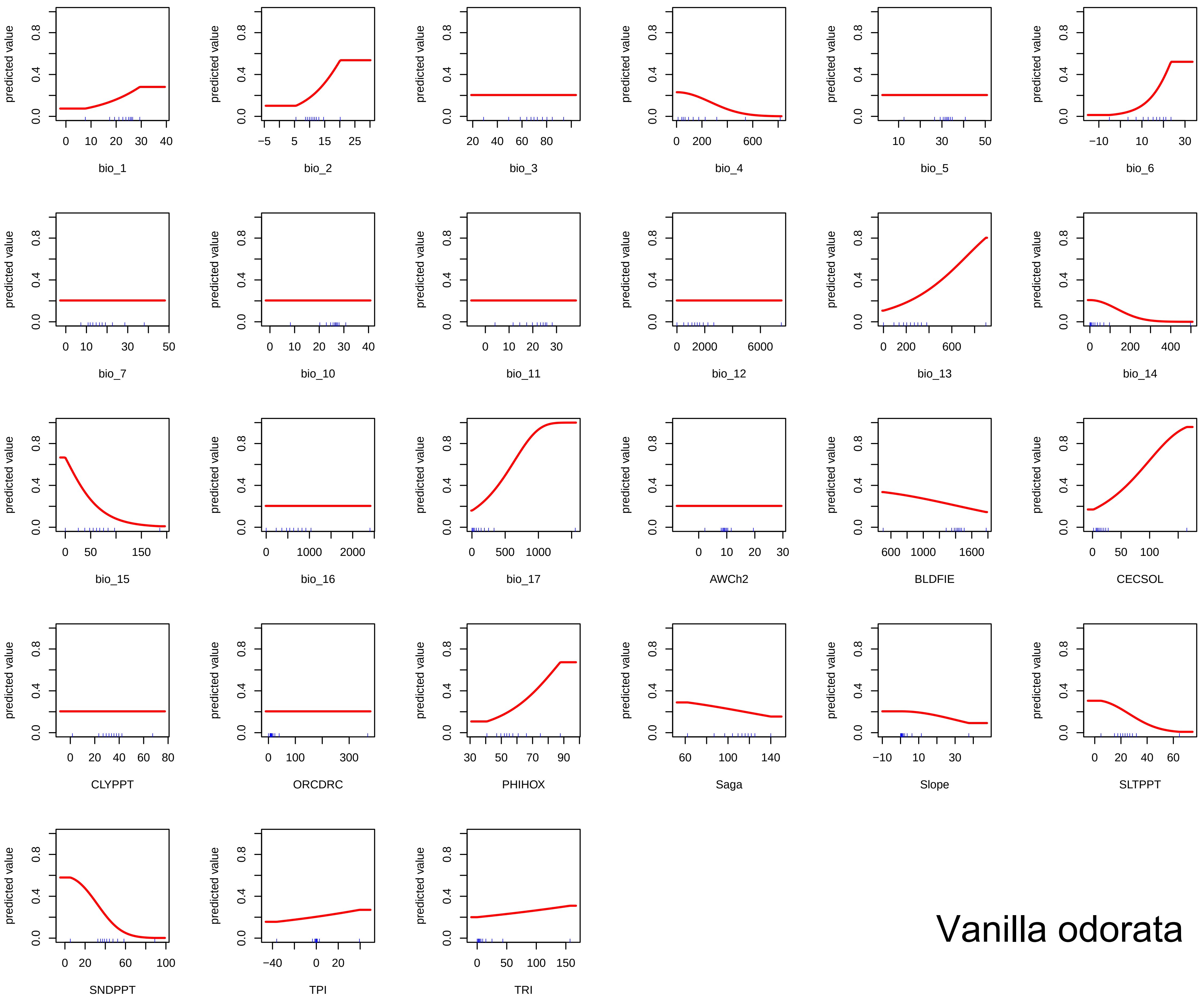
predicted value
bio_1
bio_2
bio_3
bio_4
bio_5
bio_6
bio_7
bio_10
bio_11
bio_12
bio_13
bio_14
bio_15
bio_16
bio_17
AWCh2
BLDFIE
CECSOL
CLYPPT
ORCDRC
PHIHOX
Saga
Slope
SLTPPT
SNDPPT
TPI
TRI
Vanilla odorata

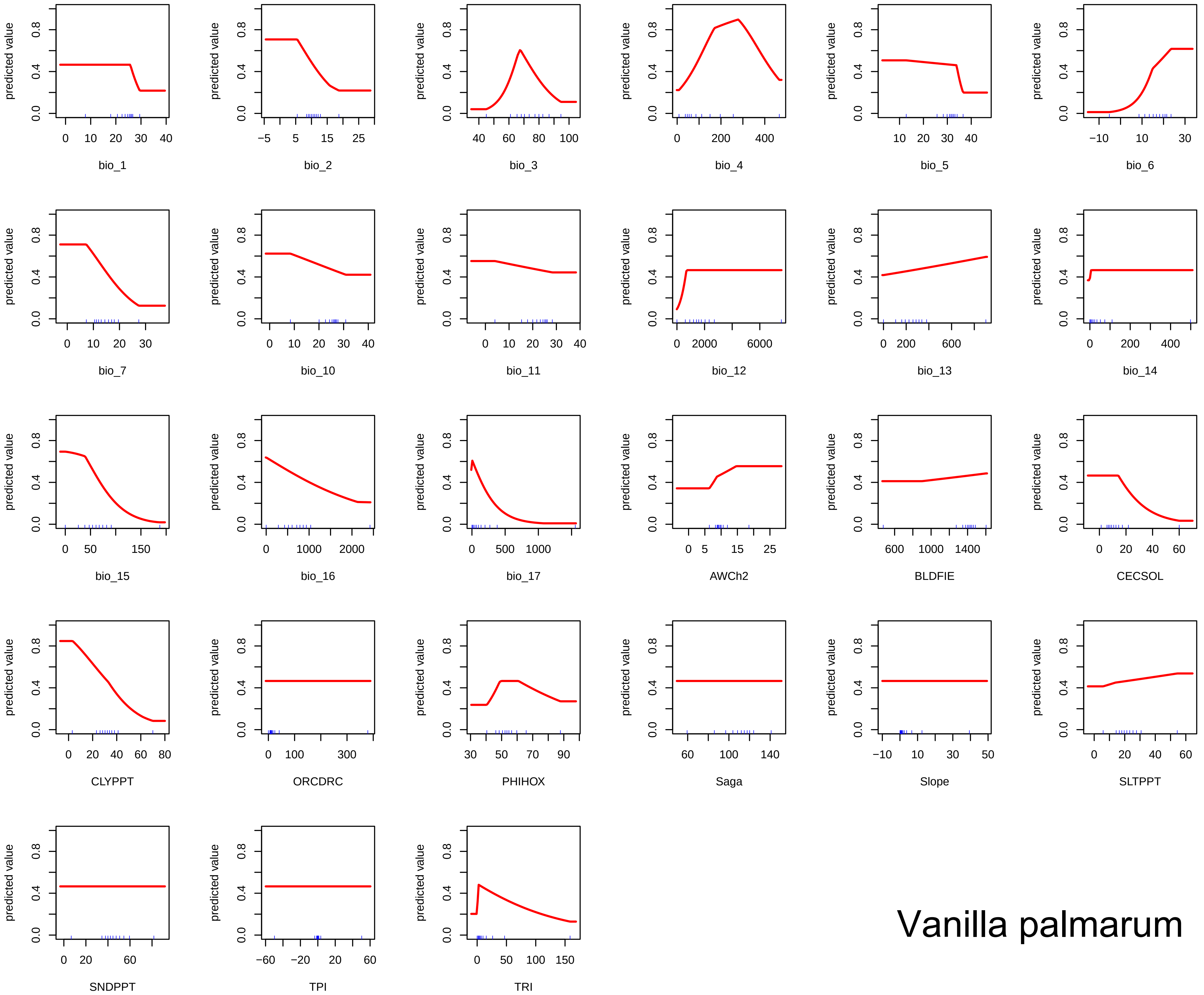
predicted value
bio_1
bio_2
bio_3
bio_4
bio_5
bio_6
bio_7
bio_10
bio_11
bio_12
bio_13
bio_14
bio_15
bio_16
bio_17
AWCh2
BLDFIE
CECSOL
CLYPPT
ORCDRC
PHIHOX
Saga
Slope
SLTPPT
SNDPPT
TPI
TRI
Vanilla palmarum

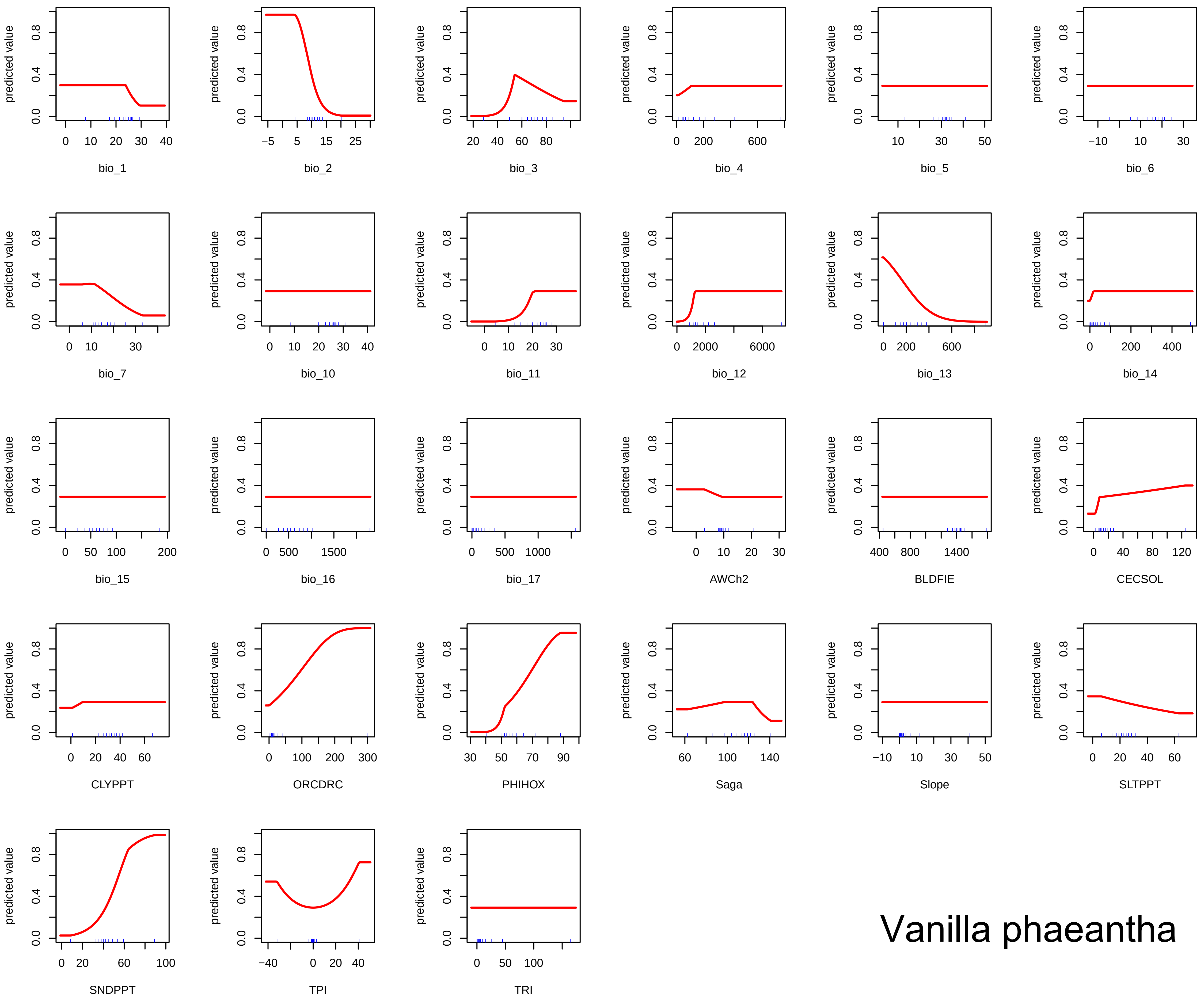
predicted value
bio_1
bio_2
bio_3
bio_4
bio_5
bio_6
bio_7
bio_10
bio_11
bio_12
bio_13
bio_14
bio_15
bio_16
bio_17
AWCh2
BLDFIE
CECSOL
CLYPPT
ORCDRC
PHIHOX
Saga
Slope
SLTPPT
SNDPPT
TPI
TRI
Vanilla phaeantha

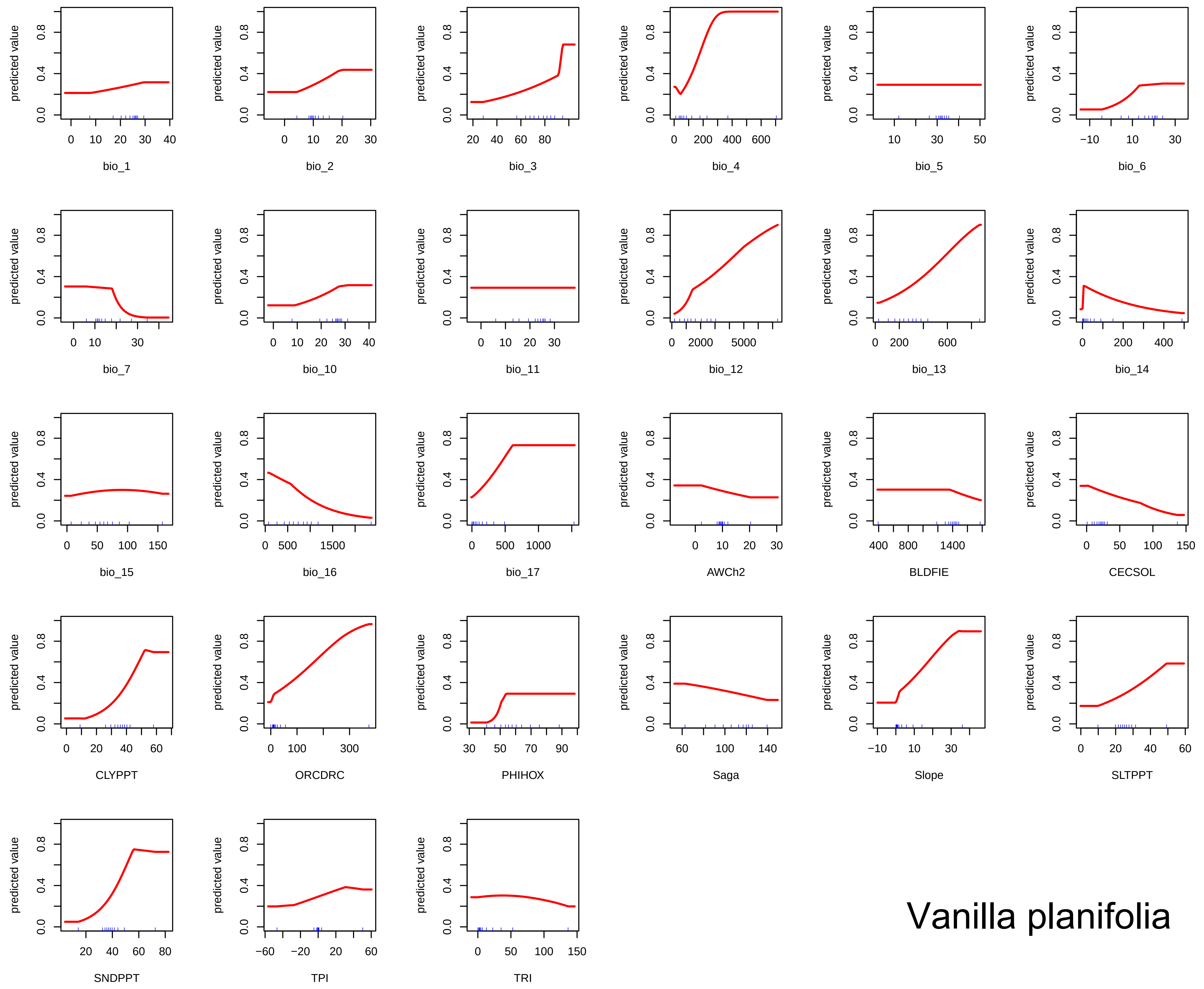
predicted value
bio_1
bio_2
bio_3
bio_4
bio_5
bio_6
bio_7
bio_10
bio_11
bio_12
bio_13
bio_14
bio_15
bio_16
bio_17
AWCh2
BLDFIE
CECSOL
CLYPPT
ORCDRC
PHIHOX
Saga
Slope
SLTPPT
SNDPPT
TPI
TRI
Vanilla planifolia

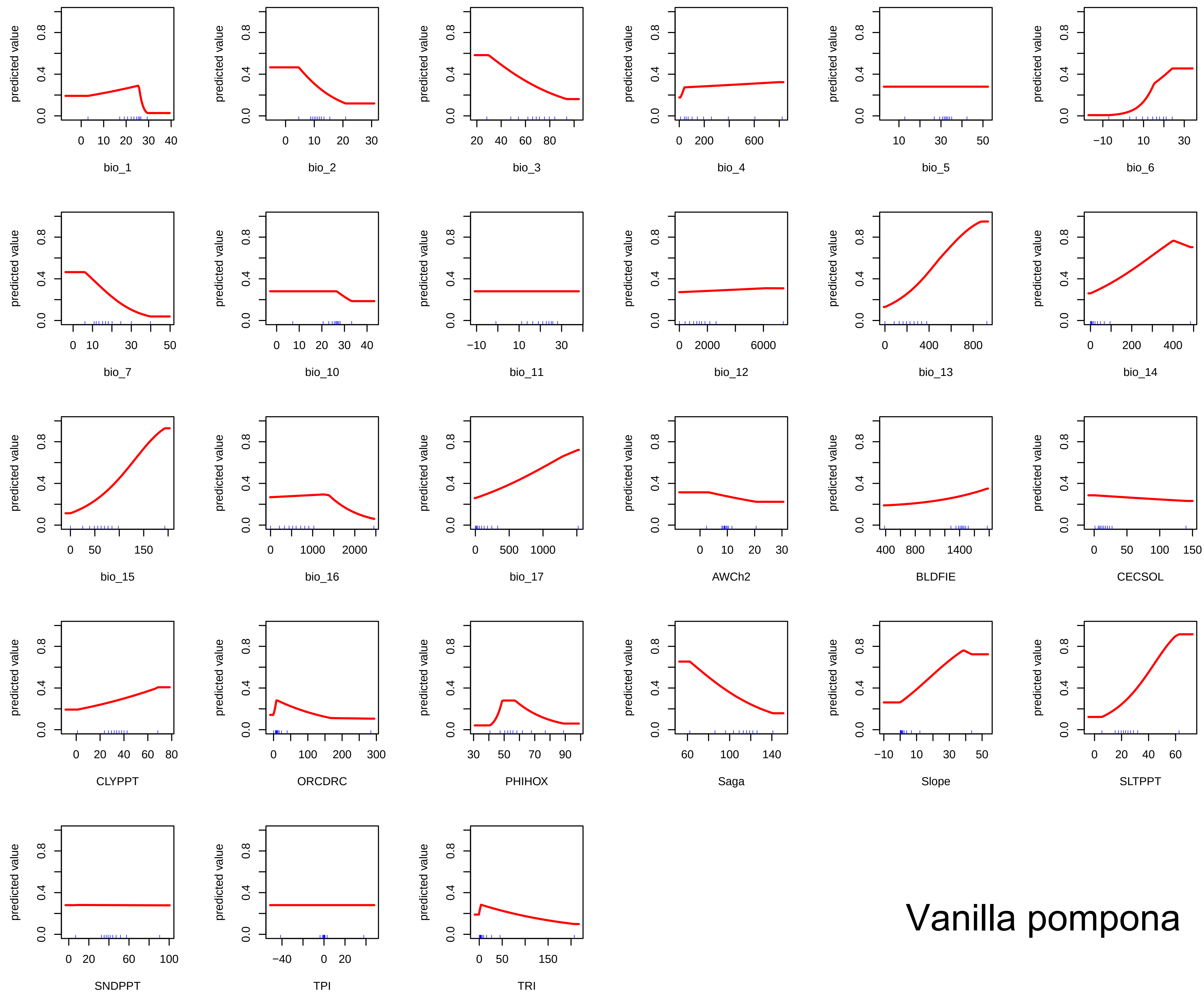
predicted value
0.0
0.4
0.8
bio_1
bio_2
bio_3
bio_4
bio_5
bio_6
bio_7
bio_10
bio_11
bio_12
bio_13
bio_14
bio_15
bio_16
bio_17
AWCh2
BLDFIE
CECSOL
CLYPPT
ORCDRC
PHIHOX
Saga
Slope
SLTPPT
SNDPPT
TPI
TRI
Vanilla pompona

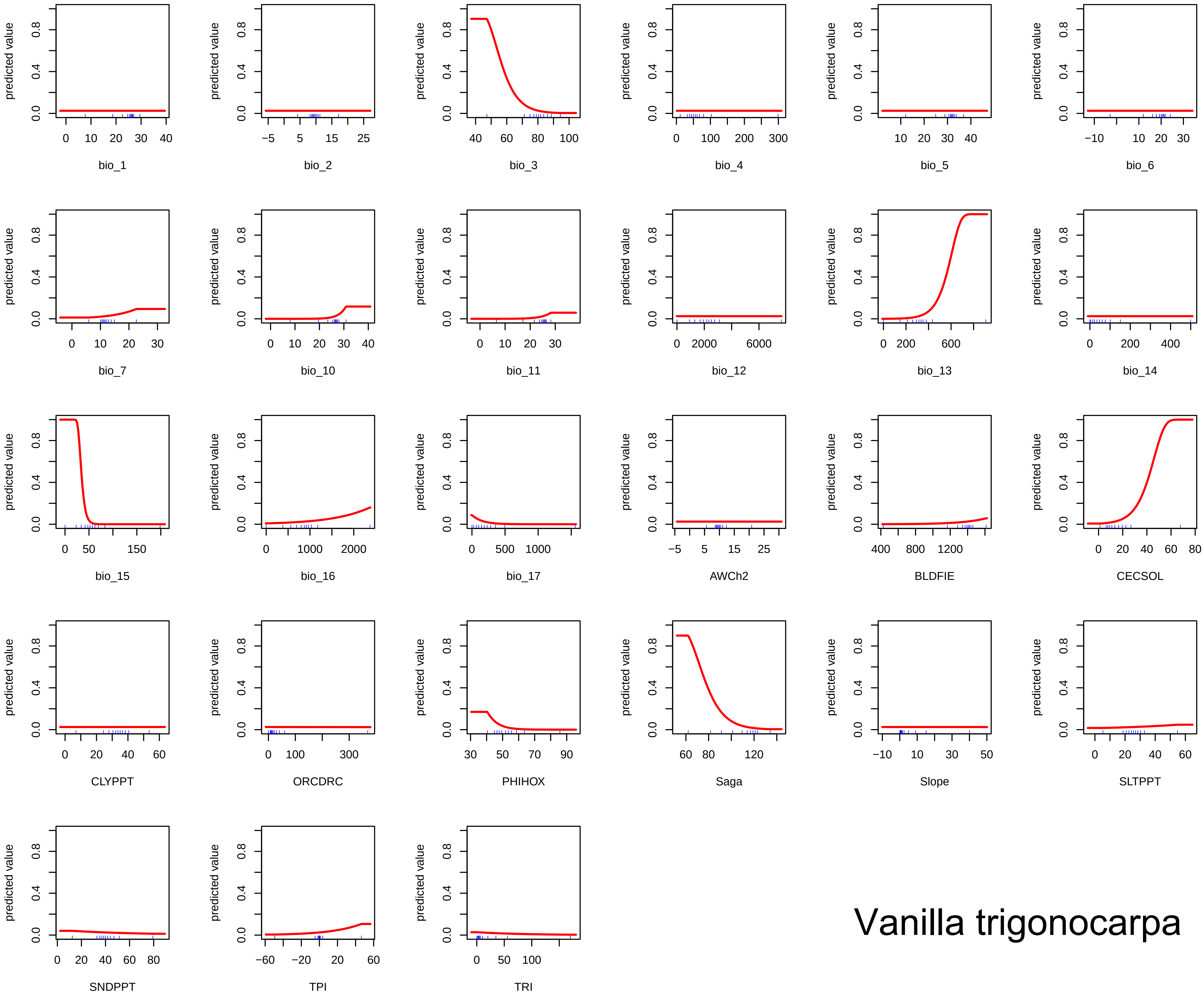
predicted value
bio_1
bio_2
bio_3
bio_4
bio_5
bio_6
bio_7
bio_10
bio_11
bio_12
bio_13
bio_14
bio_15
bio_16
bio_17
AWCh2
BLDFIE
CECSOL
CLYPPT
ORCDRC
PHIHOX
Saga
Slope
SLTPPT
SNDPPT
TPI
TRI
Vanilla trigonocarpa
